# The impairment of HCCS leads to MLS syndrome by activating a non-canonical cell death pathway in the brain and eyes

**DOI:** 10.1002/emmm.201201739

**Published:** 2013-01-22

**Authors:** Alessia Indrieri, Ivan Conte, Giancarlo Chesi, Alessia Romano, Jade Quartararo, Rosarita Tatè, Daniele Ghezzi, Massimo Zeviani, Paola Goffrini, Ileana Ferrero, Paola Bovolenta, Brunella Franco

**Affiliations:** 1 Telethon Institute of Genetics and Medicine (TIGEM)Naples, Italy; 2 Departments of Genetics, Biology of Microorganisms, Anthropology and Evolution, University of ParmaParma, Italy; 3 Integrated Microscopy Facility, Institute of Genetics and Biophysics “Adriano Buzzati Traverso”CNR, Naples, Italy; 4 Unit of Molecular Neurogenetics, The Foundation “Carlo Besta” Institute of NeurologyMilan, Italy; 5 Centro de Biología Molecular “Severo Ochoa”, CSIC-UAM and CIBER de Enfermedades Raras (CIBERER)Madrid, Spain; 6 Medical Genetics Services, Department of Translational Medical Sciences, Federico II UniversityNaples, Italy

**Keywords:** apoptosis, eye development, holo-cytochrome c-type synthase, mitochondrial diseases, MLS syndrome

## Abstract

Mitochondrial-dependent (intrinsic) programmed cell death (PCD) is an essential homoeostatic mechanism that selects bioenergetically proficient cells suitable for tissue/organ development. However, the link between mitochondrial dysfunction, intrinsic apoptosis and developmental anomalies has not been demonstrated to date. Now we provide the evidence that non-canonical mitochondrial-dependent apoptosis explains the phenotype of microphthalmia with linear skin lesions (MLS), an X-linked developmental disorder caused by mutations in the holo-cytochrome *c*-type synthase (*HCCS*) gene. By taking advantage of a medaka model that recapitulates the MLS phenotype we demonstrate that downregulation of *hccs*, an essential player of the mitochondrial respiratory chain (MRC), causes increased cell death via an apoptosome-independent caspase-9 activation in brain and eyes. We also show that the unconventional activation of caspase-9 occurs in the mitochondria and is triggered by MRC impairment and overproduction of reactive oxygen species (ROS). We thus propose that HCCS plays a key role in central nervous system (CNS) development by modulating a novel non-canonical start-up of cell death and provide the first experimental evidence for a mechanistic link between mitochondrial dysfunction, intrinsic apoptosis and developmental disorders.

## INTRODUCTION

The microphthalmia with linear skin lesions syndrome (MLS, OMIM 309801) is a rare X-linked dominant neuro-developmental disorder caused by mutations in the *HCCS* gene (Wimplinger et al, 2006). Our recent work has also implicated *COX7B* in the pathogenesis of this disorder (Indrieri et al, 2012). MLS is characterized by unilateral or bilateral microphthalmia and linear skin defects, limited to the face and neck in affected females and in utero lethality in males. Although *HCCS* is ubiquitously expressed (Schaefer et al, 1996; Schwarz & Cox, 2002), the highly specific clinical features observed in MLS affected females suggest a critical function and a sensitive dosage for HCCS in the eye and the skin. Microcephaly, mental retardation, diaphragmatic hernia and congenital heart defects are additional features less frequently observed in female patients (Morleo & Franco, 2009 [update 2011]; Sharma et al, 2008), implying that non-random X chromosome inactivation may also influence the MLS phenotype (Franco & Ballabio, 2006; Morleo & Franco, 2008; Van den Veyver, 2001).

*HCCS* is a highly conserved gene from fungi to metazoans, and encodes a mitochondrial holo-cytochrome c (Cytc)-type synthase, also known as ‘heme lyase’, located on the outer surface of the inner mitochondrial membrane (Schaefer et al, 1996; Schwarz & Cox, 2002). HCCS catalyses the covalent attachment of heme to both Cytc and Cytc_1_, which are crucial components of the mitochondrial respiratory chain (MRC): Cytc_1_ (an integral component of complex III) transfers the electrons to Cytc, which, in turn, transfers the electrons from complex III to complex IV (Smeitink et al, 2001). This class of heme lyases was first identified in *Saccharomyces cerevisiae*. In yeast, the heme incorporation into Cytc and Cytc_1_ is carried out by two distinct proteins, encoded by the two genes *Cyc3* and *Cyt2*, respectively. Inactivation of each gene product results in the loss of respiratory growth (Dumont et al, 1987; Zollner et al, 1992). A single heme lyase, HCCS, is instead required for maturation of both Cytc and Cytc_1_ in higher eukaryotes (Bernard et al, 2003; Schaefer et al, 1996).

The features of MLS syndrome differ from those usually found in mitochondrial diseases. For instance, HCCS and COX7B are the only human MRC-related proteins, the impairment of which causes microphthalmia. Interestingly, functional studies in mammals revealed a tissue/cell type-specific requirement for the ubiquitous HCCS protein. In injured adult rat motor neurons, apoptotic stimuli induce Hccs translocation outside the mitochondria resulting in suppression of the X-linked inhibitor of apoptosis (XIAP) protein, and subsequent cell-death activation (Kiryu-Seo et al, 2006). Conditional inactivation of *Hccs* in mouse heart results in severe defects of the MRC and in the accumulation of massively enlarged and aberrant mitochondria, with disorganized cristae, leading to mid-gestational lethality in hemizygous *Hccs* knockout (KO) males. Notably, heterozygous *Hccs* KO females show very slow rate of cardiomyocyte proliferation during embryonic development with no changes in the rate of cell death (Drenckhahn et al, 2008).

However, these findings do not explain the precise role/s of HCCS in the pathogenesis of the developmental defects observed in MLS syndrome, *i.e.* microphthalmia and microcephaly.

During central nervous system (CNS) development programmed cell death (PCD) represents an important mechanism regulating the size of cell populations although the mechanisms that regulate the survival/death decision are not fully characterized. In particular, retinal cells are generated from a pool of progenitor cells that exit the cell cycle and acquire specific cell fates with a precise spatial and temporal order that depends on both intrinsic and extrinsic factors (Livesey & Cepko, 2001; Marquardt & Gruss, 2002). Overproduced retinal cells are selectively eliminated in a series of ordered apoptotic waves that contribute to determine the final size of the retinal neuroepithelium (Valenciano et al, 2009; Vecino et al, 2004). Interestingly, in the retina as well as in other CNS structures, cell death largely occurs through the activation of the mitochondrial-dependent (or intrinsic) apoptotic pathway, which is thus a crucial cascade for proper eye development (Guerin et al, 2006; Isenmann et al, 2003; Laguna et al, 2008). In the intrinsic pathway, mitochondrial outer membrane permeabilization leads to the release of Cytc and other proapoptotic proteins in the cytosol thus driving the initiator caspase activation and cell death (Tait & Green, 2010).

In this study, we better defined HCCS biochemical function and the effects of its pathological mutations on the mitochondrial oxidative phosphorylation (OXPHOS) using a yeast *in vitro* model. In addition, we exploited the medaka fish (*Oryzias latipes*) as a model system to investigate the role of HCCS in MLS syndrome, focusing on the role of the *hccs* medaka homolog in eye development. We show that *hccs* knockdown in medaka recapitulates the phenotype observed in MLS syndrome. We present evidence that the hccs-dependent microphthalmic and microcephalic phenotype is due to a mitochondrial caspase-9 activation that occurs in an apoptosome-independent manner. We also show that caspase-9 activation and subsequent retinal cell death are the consequence of mitochondrial respiratory malfunction and overproduction of reactive oxygen species (ROS).

Taken together, our results demonstrate that HCCS plays a key role during CNS development by controlling a non-canonical mitochondrial-dependent PCD pathway via activation and release of caspase-9 from mitochondria. Thus, in addition to controlling holo-Cytc synthesis, HCCS regulate the activation of an unconventional pathway leading to cell death in an apoptosome-independent manner.

## RESULTS

### HCCS deficiency impairs mitochondrial oxidative phosphorylation and affects cell survival in yeast

HCCS is highly conserved from yeast to humans. Thus, in order to define HCCS biochemical function and its effect on OXPHOS, we performed complementation studies in B-8025-Δ*cyc3*, a *S. cerevisiae* strain deficient in the HCCS orthologous gene product Cyc3. The B-8025-Δ*cyc3* strain is unable to grow in non-fermentable carbon sources as expected for a strain with a severely depleted respiratory function (Dumont et al, 1987). Transformation of B-8025-Δ*cyc3* with the human *HCCS* restored mitochondrial respiration and oxidative growth, unlike three forms bearing mutations identified in MLS patients. These include two missense mutations (E159K; R217C) involving two highly conserved amino acids and a non-sense mutation (Δ197-268) resulting in a HCCS-protein lacking 72 c-terminal amino acids that are required for mitochondrial targeting (Wimplinger et al, 2006, 2007). In order to assess the structural integrity of the MRC complexes we analysed the content of mitochondrial cytochromes by measuring their absorption spectra. The spectrum profile of the B-8025-Δ*cyc3* strain expressing the human *HCCS* gene was indistinguishable from that of the wt yeast strain (B-7553; Supporting Information Fig S1A). On the contrary, the B-8025-Δ*cyc3* strain expressing the *HCCS* mutant alleles, or the pYEX empty vector, presented a pronounced reduction of the Cytc absorption peak and the absence of the spectrum for Cytaa3, a component of complex IV (Supporting Information Fig S1A). This indicates the loss of the structural integrity of complex IV that requires the presence of the folded and mature holo-Cytc (Pearce & Sherman, 1995). The reduction in cytochromes content was paralleled by a decrease in the respiratory activity of the B-8025-Δ*cyc3* strain expressing either the *HCCS* mutant alleles or the pYEX empty vector (Supporting Information Fig S1B), indicating that the mutated *HCCS* alleles identified in MLS patients are pathogenic via impaired OXPHOS.

We then analysed the amount of Cytc in the mitochondria of the B-8025-Δ*cyc3* strain expressing the *HCCS* mutant alleles or the pYEX empty vector. Cytc is encoded by a nuclear gene and lacks a canonical amino-terminal mitochondrial targeting sequence. According to the current model, heme-less apo-Cytc is freely transported across the outer mitochondrial membrane (OMM) into the mitochondrial intermembrane space, where it binds to HCCS and is trapped by an irreversible conversion to heme-containing holo-Cytc (Dumont et al, 1991; Mayer et al, 1995). In agreement with these observations, the levels of Cytc in the B-8025-Δ*cyc3* strain were strongly reduced (Supporting Information Fig S1C). Notably, the E159K allele had a partial effect in rescuing the mitochondrial levels of Cytc although the spectrum profiles of both E159K and R217C were similarly compromised (Supporting Information Fig S1A and C). This result indicates that whilst both mutant HCCS proteins fail to attach the heme moiety into apo-Cytc, the R217C protein prevents translocation of Cytc, whereas the E159K is permissive, suggesting that the import of the heme-less apo-Cytc into mitochondria requires the presence of the HCCS protein even if not catalytically active. These data point to an additional role of HCCS as an apo-Cytc chaperone; the differences between E159K and R217C mutants consist in their ability to physically bind, chaperone and drive apo-Cytc to the inner mitochondrial membrane (IMM). These results also indicate that HCCS directly controls the import of Cytc into mitochondria independently from its heme lyase activity, clearly demonstrating that HCCS binding to apo-Cytc, rather than heme attachment, is the critical step for Cytc import into mitochondria. The observed impairment of the MRC was accompanied by a decrease in yeast chronological life span (CLS) in both B-8025-Δ*cyc3* alone or expressing the *HCCS* pathological alleles (Supporting Information Fig S1D) indicating that loss of functional HCCS is deleterious for cell survival.

### *hccs* knockdown in medaka fish recapitulates the MLS syndrome

**Figure 1:**
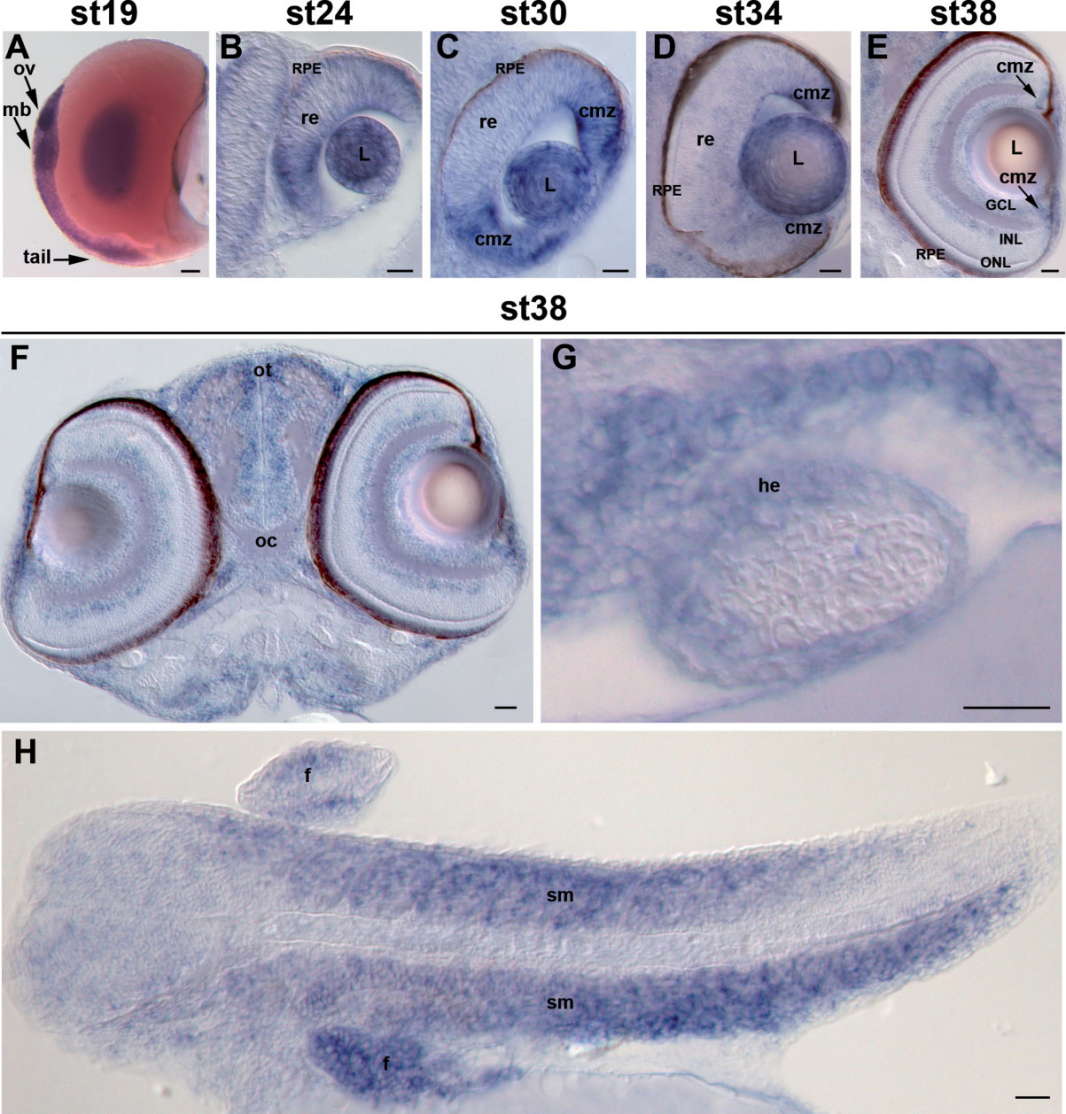
Expression pattern of *hccs* in medaka *In situ* hybridization analysis of *hccs* **A.** *hccs* expression in whole embryos at st19. Scale bar: 100 µm.**B–E.** Frontal sections of embryos at st24, st30, st34 and st38 showing a strong *hccs* expression in the different structures of the developing eye.**F, G.** Frontal sections of embryos at st38 showing the expression of *hccs* in the central nervous system and in the heart, respectively.**H.** Sagittal section of embryos at st38 showing a strong *hccs* expression in the skeletal muscles. Scale bars: 20 µm. ov, optic vesicle; mb, midbrain; re, retina; L, lens; cmz, ciliary marginal zone; GCL, ganglion cell layer; INL, inner nuclear layer; ONL, outer nuclear layer; RPE, retinal pigmented epithelium; ot, optic tectum; oc, optic chiasm; he, heart, sm, skeletal muscles; f, fin.

Next, to gain insight into the mechanism of defective organ development in MLS, we investigated a vertebrate animal model. Genetic inactivation of *Hccs* results in ES cell lethality in the mouse (Prakash et al, 2002), preventing the generation of a suitable mammalian model for MLS syndrome. We thus turned to the teleost medaka fish in which a morpholino (MO)-based knockdown approach enables the generation of morphant embryos with less dramatic phenotypes (Wittbrodt et al, 2002). Publicly available sequences were used to identify the *HCCS* homolog in medaka (*hccs*; see Supporting Information Materials and Methods section and Supporting Information Fig S2). RNA *in situ* hybridization (ISH) studies revealed that *hccs* is ubiquitously expressed at all developmental stages analysed, with higher expression levels in the eyes, CNS, heart and skeletal muscle (Fig 1). In particular, at stage (st) 19 we found high levels of expression in the optic vesicle, the presumptive retinal pigmented epithelium (RPE), the presumptive mesencephalic region and the tail (arrows in Fig 1A). At st24 high levels of expression were detected in the lens placode, the RPE and the neural retina of the optic cup, with an apparent peripheral^high^ to central^low^ gradient (Fig 1B). At later stages (st30, st34, st38), *hccs* is also expressed in the retinal progenitor cells within the ciliary marginal zone as well as in the ganglion and amacrine cells (Fig 1C–E). At st38, similarly to what reported in human and mouse (Ramskold et al, 2009; Schaefer et al, 1996; Schwarz & Cox, 2002), high expression levels were found in the CNS (Fig 1F), the heart (Fig 1G) and the skeletal muscles (Fig 1H).

**Figure 2:**
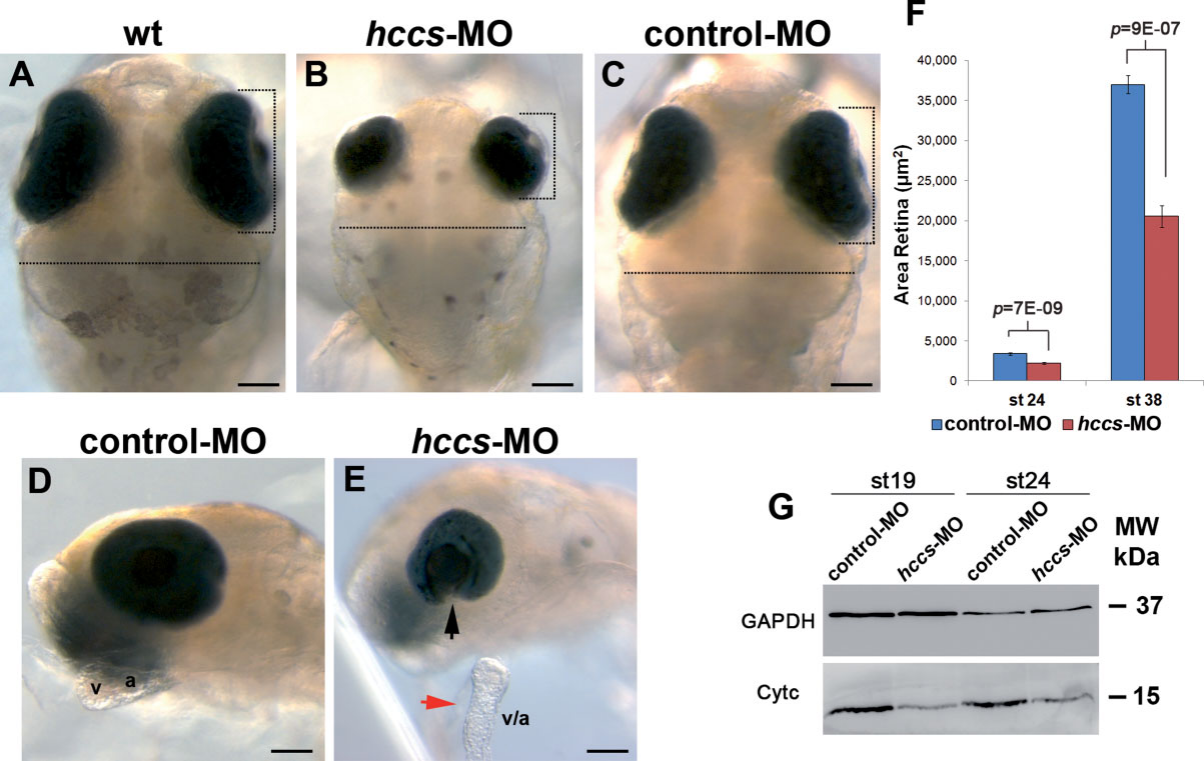
Knock-down of *hccs* in medaka recapitulates the phenotypic features of MLS syndrome **A–E.** Bright-field dorsal (**A–C**) and lateral (**D**, **E**) views of wt (**A**), *hccs*-MO (**B**, **E**) and control-MO (**C**, **D**) -injected embryos at st38. *hccs*-morphants display microphthalmia (vertical dashed line in **B**), microcephaly (horizontal dashed line in **B**) and cardiac defects including failure of heart loop formation and pericardial oedema (red arrow in **E**). In a significant number of the microphthalmic embryos (50%), RPE layering and closure of ventral optic fissure were also impaired resulting in coloboma formation (black arrow in **E**; see also Supporting Information Table S1). Embryos injected with control-MO did not show any abnormal phenotype (**C**, **D**). a, atrium; v, ventricle. Scale bars: 100 µm.**F.** Analysis of the eye size at st24 and st38 (error bars are SEM; *n* ≥ 19 eyes, *p*-values were calculated by two-tailed Student's *t*-test).**G.** Western blotting analysis revealed decreased levels of total Cytc in morphants (*hccs*-MO) at st19 and st24 compared to control embryos (control-MO). GAPDH was used as loading control.

To determine the function of *hccs* during embryonic development and its role in the onset and progression of MLS syndrome, we designed two specific morpholinos (MO), one directed against the ATG region of *hccs* (*hccs*-MO), the second against the third splice donor site (*hccs*-MO2). Embryos injected with *hccs*-MO developed normally until optic-cup stage, when they begun to display a very frequent (70 ± 5% of 3000 injected embryos) and morphologically recognizable microphthalmia (vertical dashed line in Fig 2B and E). These defects were associated with microcephaly (horizontal dashed line in Fig 2B) and cardiovascular abnormalities including failure of heart loop formation and pericardial oedema (red arrow in Fig 2E) and death at hatching stage (st39). Furthermore, RPE layering and closure of ventral optic fissure were impaired resulting in coloboma formation in 50% of microphthalmic embryos (black arrow in Fig 2E). A small fraction of embryos (20 ± 5%) displayed a more severe phenotype with anophthalmia and/or microphthalmia associated with severe trunk defects, whereas the remaining embryos (10 ± 5%) died before gastrulation (see Supporting Information Table S1). Embryos injected with *hccs*-MO2 showed the same phenotype (Supporting Information Fig S3B and Table S1). Since no phenotypic differences were observed between embryos injected with *hccs*-MO or *hccs*-MO2, subsequent studies were performed exclusively with the *hccs*-MO. The efficiency and specificity of the selected MO were further verified using a series of recommended controls (Eisen & Smith, 2008; Robu et al, 2007; see Supporting Information Materials and Methods section, Supporting Information Fig S3 and Table S1). A mutated form of the *hccs*-MO (control-MO, see Supporting Information Materials and Methods section and Supporting Information Table S1) was used as control.

Biochemical analysis of MRC complexes in hccs-deficient medaka embryos revealed a significant reduction of complex III activity whereas those of complexes I, II and IV were normal (Supporting Information Fig S4). Using Western blotting analysis, we detected also a decrease in the levels of total (Fig 2G) and mitochondrial (Supporting Information Fig S5) Cytc in morphant embryos, similarly to what demonstrated in the yeast. These data indicate impaired mitochondrial respiration in hccs-deficient embryos.

As expected from an OXPHOS-deficient tissue, transmission electron microscopy (TEM) analysis showed the presence of abnormal mitochondria, with internal disorganization of the cristae in morphant retinas (Supporting Information Fig S6), further supporting the key role of hccs in the mitochondrial physiology of vertebrates.

### Caspase-dependent cell death underlies microphthalmia in hccs-deficient embryos

**Figure 3:**
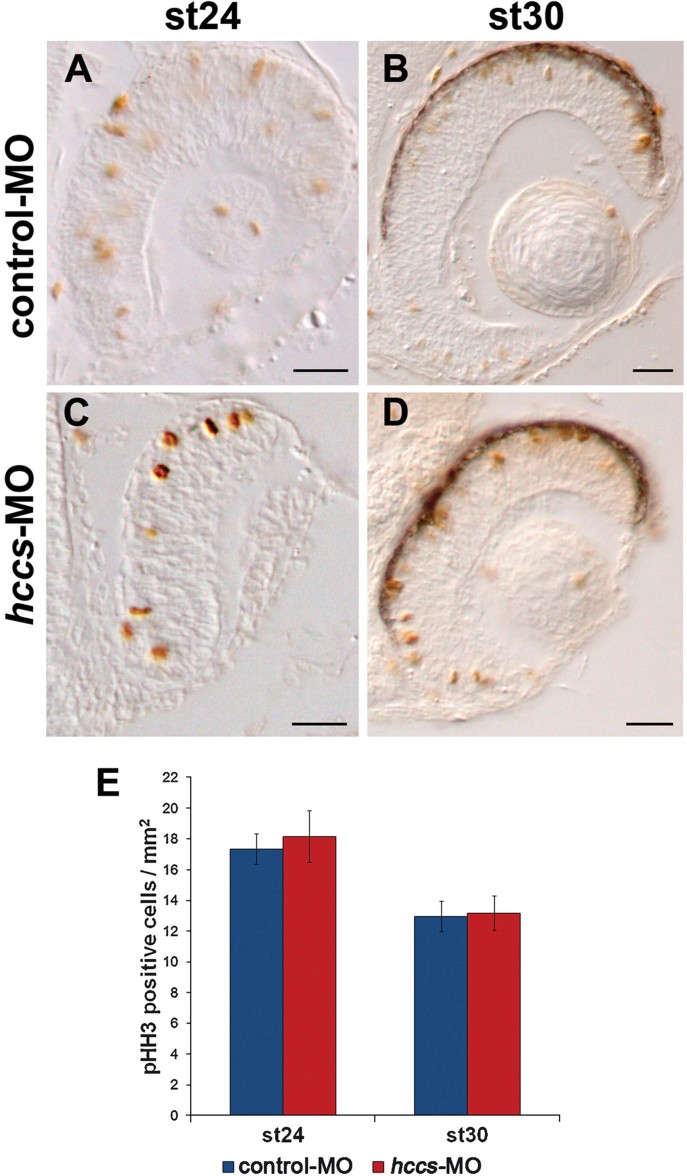
Analysis of cell proliferation in *hccs*-morphant embryos **A–D.** Immunohistochemistry with α-pHH3. Embryos injected with control-MO (**A**, **B**) and with *hccs*-MO (**C**, **D**). Scale bars: 20 µm.**E.** Number of pHH3-positive cells normalized for area (mm^2^). At st24 and st30 morphant embryos do not show abnormalities in cell proliferation (*n* ≥ 5 embryos/stage, error bars are SEM).

Heart-specific *Hccs* inactivation delays cardiomyocyte proliferation in the mouse (Drenckhahn et al, 2008), and HCCS and Cytc have been implicated in caspase-dependent PCD (Jiang & Wang, 2004; Kiryu-Seo et al, 2006). Thus, we determined whether alterations in proliferation and/or apoptosis in *hccs*-morphants could account for the microphthalmia and the other observed CNS abnormalities. Immunostaining for phosphorylated histone H3 (pHH3), a specific marker for cells in M-phase, revealed no significant differences between the number of proliferating cells in the neural retina and RPE of morphants *versus* control embryos neither at st24, when the ocular phenotype can first be detected, nor at st30 (Fig 3A–E) when the microphthalmic phenotype is evident.

**Figure 4:**
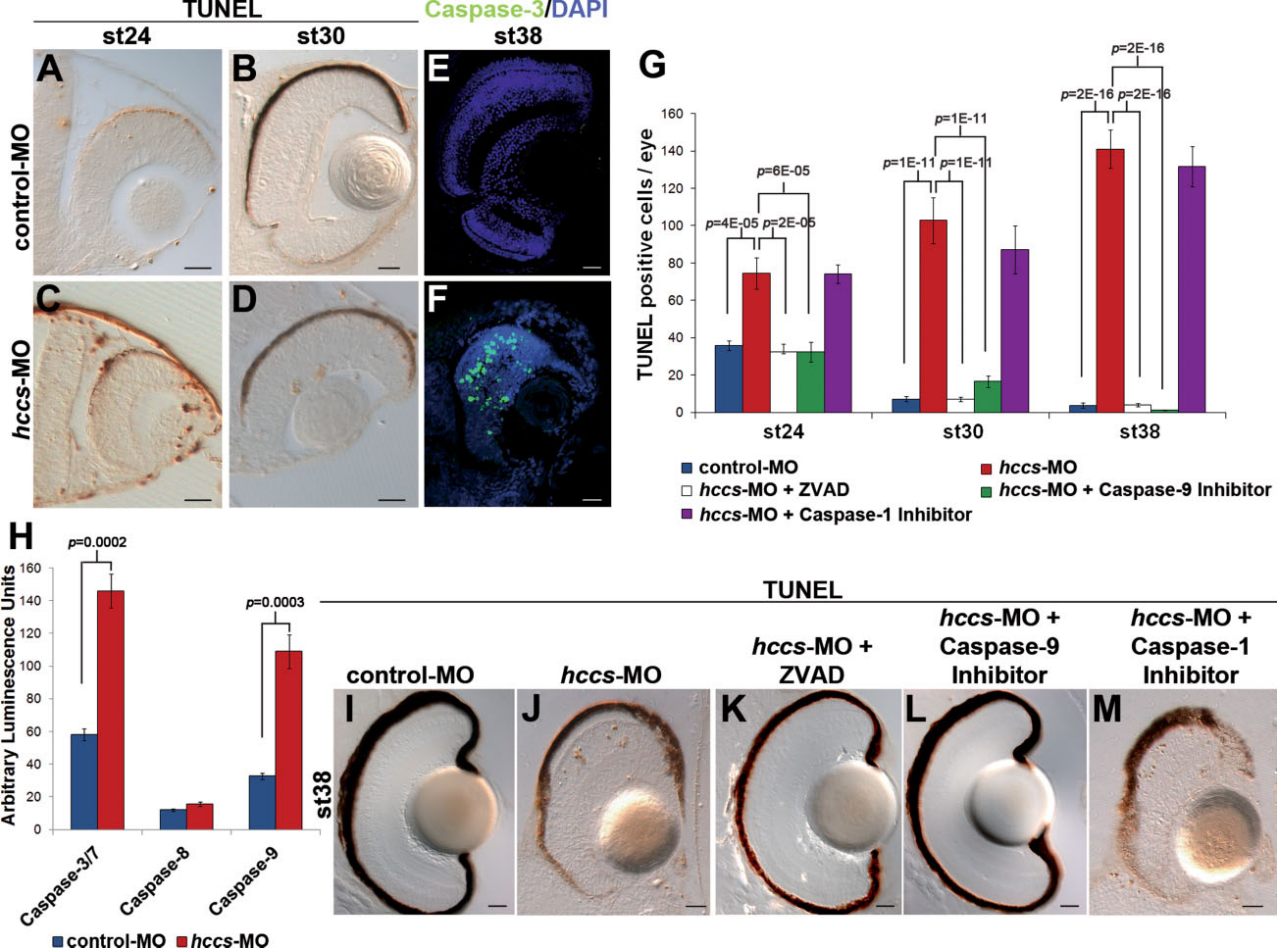
Caspase-9 dependent cell death underlies microphthalmia in hccs-deficient embryos **A–D.** TUNEL assays on retinal sections. Embryos injected with control-MO (**A**, **B**) and with *hccs*-MO (**C**, **D**) at st24 and st30.**E, F.** Immunofluorescence analysis with an anti-active-caspase-3 antibody on control-MO (**E**) and *hccs*-MO injected embryos (**F**) at st38.**G.** Number of TUNEL positive cells/eye (*n* ≥ 5 embryos/stage; Error bars are SEM; *p*-values were calculated by ANOVA).**H.** Caspase activation in control and morphant embryos measured by cleavage of a synthetic substrate and release of luciferin (DEVD-aminoluciferin for caspase-3, LETD-aminoluciferin for caspase-8, and LEHD-aminoluciferin for caspase-9). Histograms show the relative levels of emitted signals displayed in arbitrary units of luminescence. Values represent means of four samples. Each sample represents a group of 20 embryos (error bars are SEM; *p*-values were calculated by unpaired, two-tailed Student's *t*-test).**I–M.** TUNEL assays on st38 embryos injected with control-MO (**I**) and *hccs*-MO alone (**J**), in association with the pan-caspase inhibitor (ZVAD) (**K**) or with the caspase-9 inhibitor (**L**), or with the caspase-1 inhibitor (**M**) (*n* = 100 embryos for each treatment). The pan-caspase inhibitor and the specific caspase-9 inhibitor rescue the increased levels of cell death (**G**, **K**, **L**). Scale bars: 20 µm.

The spatio-temporal distribution of developmental cell death has been well characterized in medaka CNS (Iijima & Yokoyama, 2007). In good agreement with this report, in the retina of control embryos we detected the highest peak of TUNEL-positive cells at st24, whereas only occasional apoptotic cells were detected thereafter (Fig 4A and G). In contrast, the retinas of *hccs*-morphants not only displayed a significant increase in the number of TUNEL-positive cells at st24, but were also characterized by a sustained apoptosis at later stages of development coinciding with the onset of a visible microphthalmia (Fig 4A–D and G).

Upon apoptotic stimuli, Hccs inhibits the activity of the XIAP protein, leading to caspase-3 activation in the cytosol (Kiryu-Seo et al, 2006). Holo-Cytc is a crucial mediator of mitochondrial-dependent PCD. Following its release from mitochondria, Cytc binds Apaf1 in the cytosol and recruits and activates the initiator caspase-9 to form the apoptosome complex (Li et al, 1997; Rodriguez & Lazebnik, 1999). The presence of heme-containing holo-Cytc is necessary for this process (Martin & Fearnhead, 2002). Thus, hccs-deficient cells should be unable to activate the canonical intrinsic-apoptotic pathway owing to the absence of Cytc (Li et al, 2000). Unexpectedly, however, interference with *hccs* expression did not prevent caspase-3 activation, because active-caspase-3 positive cells were observed in the retina of morphant embryos (Fig 4E and F). In addition, exposure of *hccs*-morphants to a general caspase inhibitor (ZVAD-FMK) rescued PCD and microphthalmia (Fig 4G and K). Interestingly, increased cell death was also detected in other regions of the CNS (Supporting Information Fig S7A and B), but not in the majority of other organs including those that are affected in the MLS syndrome, such as the heart (Supporting Information Fig S7C–F).

Taken together, these results indicate that the microphthalmia and microcephaly observed in *hccs*-morphants are consequent of an increase in caspase-dependent neuronal cell death. Low levels of *hccs* expression might instead be sufficient for cell survival in other organs.

### *hccs* downregulation induces apoptosome-independent mitochondrial caspase-9 activation

PCD can be initiated by an extrinsic pathway mostly mediated by caspase-8, or by an intrinsic pathway that involves caspase-9 activation (Kumar, 2007; Riedl & Salvesen, 2007). Different experimental evidences indicate that proper retina development depends on the fine regulation of the intrinsic pathway (Guerin et al, 2006; Isenmann et al, 2003; Laguna et al, 2008). To assess the level of caspase activity in our model, we then performed an assay in which total lysates from morphant and control embryos were incubated with synthetic caspase-3/7, -9, and -8 specific substrates, conjugated to luciferin. In line with the previous results we observed an increased activation of caspase-3/7, while activity of caspase-8 was similar to that of controls (Fig 4H). Interestingly *hccs*-morphants showed also increased caspase-9 activity (Fig 4H), although the ‘canonical’ intrinsic pathway should be impaired owing to the absence of holo-Cytc (Li et al, 2000; Martin & Fearnhead, 2002). Moreover, treatment with a specific caspase-9 inhibitor significantly rescued both PCD and microphthalmia in morphant embryos whereas a caspase-1 inhibitor had no effect (Fig 4G, L and M), indicating a specific role of caspase-9 in the pathogenesis of microphthalmia in our model.

**Figure 5:**
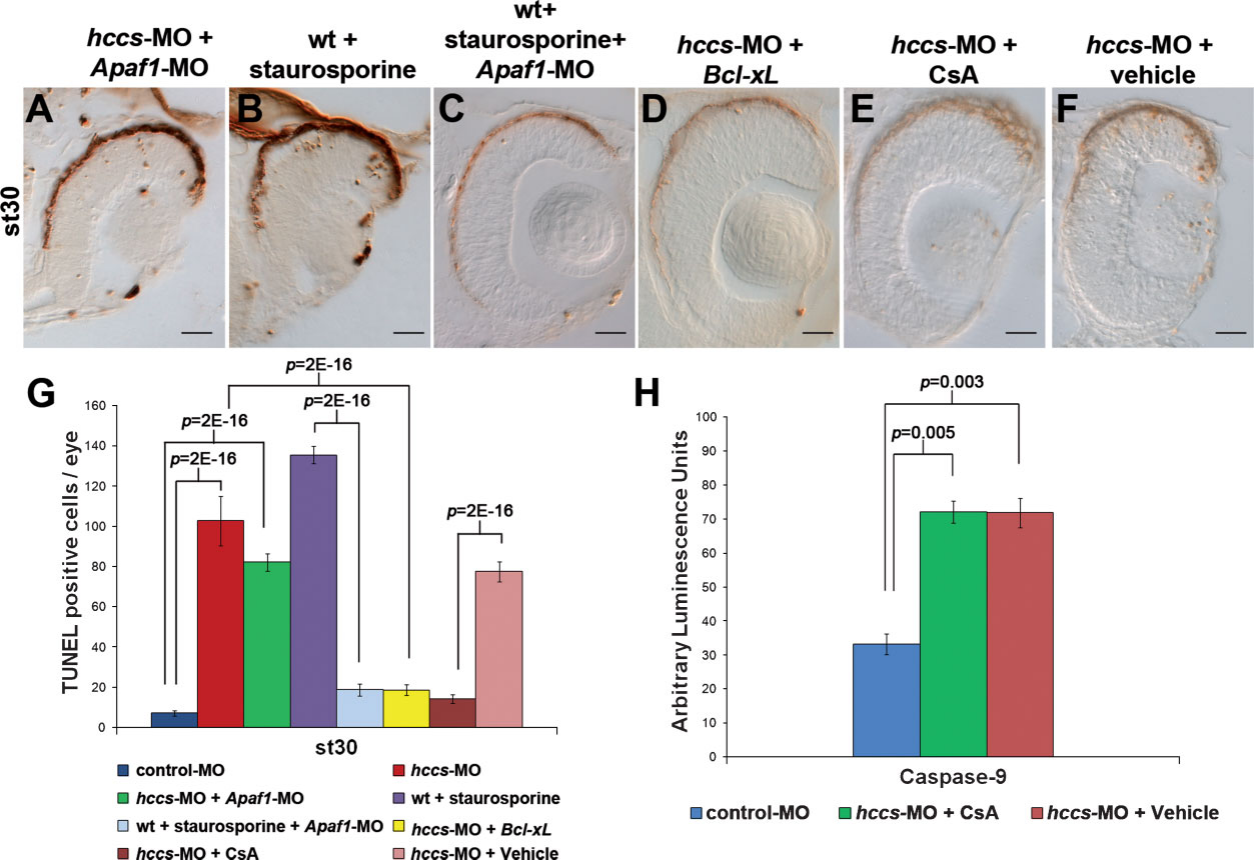
*hccs* downregulation induces apoptosome-independent caspase-9 activation in mitochondria **A–C.** TUNEL assays on st30 embryos co-injected with *hccs*-MO and *Apaf1*-MO (**A**), wt embryos exposed to staurosporine (**B**) and embryos injected with *Apaf1*-MO exposed to staurosporine (**C**) (*n* = 100/each treatment). *Apaf1* down-regulation was able to protect the embryos from cell death induced by staurosporine, but not from the increased cell death due to *hccs* knockdown.**D–F.** TUNEL assays on st30 embryos co-injected with *hccs*-MO and *Bcl-xL* mRNA (**D**) and embryos injected with *hccs*-MO treated with CsA (**E**) or vehicle alone (**F**). Note that *Bcl-xL* overexpression and CsA treatment block cell death. Scale bars: 20 µm.**G.** Number of TUNEL positive cells/eye (*n* ≥ 5 embryos/stage; Error bars are SEM; *p*-values were calculated by ANOVA).**H.** Caspase-9 activation in morphant embryos treated with CsA or vehicle alone. Histograms show the relative levels of emitted signals displayed in arbitrary units of luminescence. Values represent means of three samples. Each sample represents a group of 20 embryos (error bars are SEM; *p*-values were calculated by ANOVA).

Non-canonical apoptosome-independent caspase-9 activation has been observed in few specific conditions (Hao et al, 2005; Ho et al, 2004; Katoh et al, 2008; Manns et al, 2011; Mills et al, 2006). To determine the mechanism of caspase-9 activation in *hccs*-morphants, we thus co-injected *hccs*-MO with a MO designed against Apaf1 (*Apaf1*-MO). *Apaf1* down-regulation protected the embryos from staurosporine induced PCD but failed to rescue PCD in *hccs*-morphants. At st30, the large majority of co-injected embryos were in fact morphologically undistinguishable from *hccs*-morphants with a similar number of apoptotic cells (Fig 5A–C and G and Supporting Information Table S1). These data clearly involve non-canonical, apoptosome-independent caspase-9 activation in the pathogenesis of microphthalmia.

Caspase-9 predominantly localizes to the cytosol, although pro- and mature-caspase-9 have been also found within the mitochondria, from where they can be released into the cytosol, upon induction of apoptosis (Costantini et al, 2002; Katoh et al, 2004). Members of the Bcl-2 family can prevent this phenomenon (Costantini et al, 2002; Katoh et al, 2004). Accordingly, we observed that inhibition of OMM permeabilization, either by *Bcl-xL* overexpression or by cyclosporine A (CsA) treatment (Irwin et al, 2003; Youle & Strasser, 2008), rescued PCD in the majority of *hccs*-morphants (Fig 5D–F and G and Supporting Information Table S1). Instead, there were no significant differences in levels of caspase-9 activation between CsA-treated and vehicle-treated *hccs*-morphants (Fig 5H).

Altogether, these data strongly indicate that cell death in our model is caused by activation of caspase-9 in the mitochondria and its subsequent release in the cytosol.

### Impairment of mitochondrial functions and overproduction of ROS trigger mitochondrial caspase-9 activation leading to the MLS phenotype

**Figure 6:**
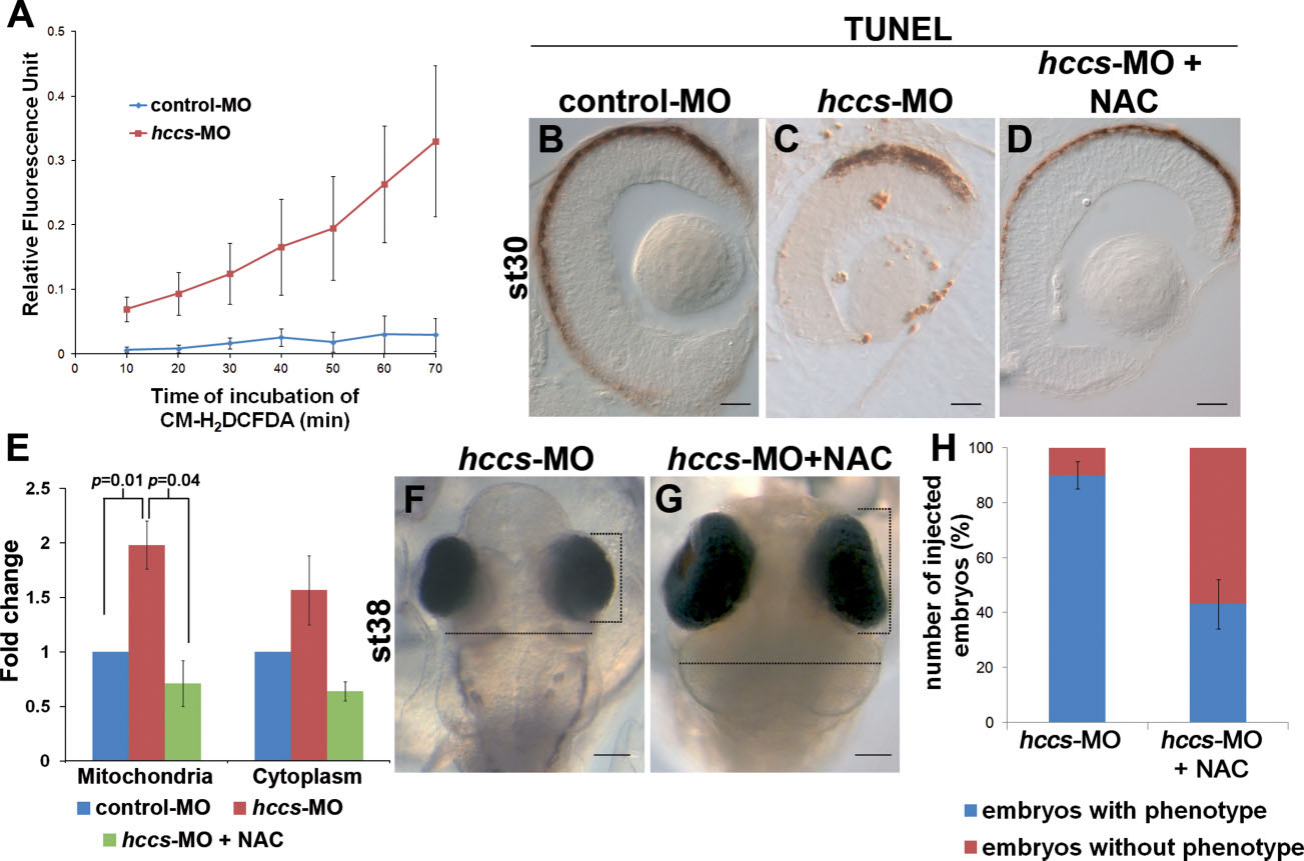
Overproduction of ROS triggers mitochondrial caspase-9 activation ultimately leading to the MLS phenotype **A.** Determination of ROS levels in *hccs*-MO injected fish. Accumulation of the CM-H_2_DCFDA dye used as indicator of ROS levels, in *hccs*-MO and control-MO injected embryos at st24. Values represent means of 10 samples. Each sample represents a group of three embryos. Error bars are SEM.**B–D.** TUNEL assays on retinal sections of st30 embryos injected with control-MO (**B**), *hccs*-MO (**C**) and *hccs*-MO treated with NAC (**D**). Note how NAC treatment blocks cell death in morphant embryos. Scale bars: 20 µm.**E.** Caspase-9 activation in mitochondrial and cytoplasmic fractions of control and morphant embryos. Histograms show the fold changes in the levels of emitted luminescence signals. Values represent means of *n* samples. Each sample represents a group of 80 embryos. Caspase-9 activation was significantly increased in the mitochondrial fractions of morphants (*n* = 4, *p* = 0.01, one-tailed Student's *t*-test) whereas the differences in cytosolic fractions were smaller with lower statistical significance (*n* = 5, *p* = 0.1, one-tailed Student's *t*-test). Note how NAC treatment significantly blocks mitochondrial caspase-9 activation (*n* = 3, *p* = 0.04, one-tailed Student's *t*-test).**F–H.** Bright-field stereomicroscopy images of *hccs*-MO (**F**) and *hccs*-MO injected embryos at st38 treated with NAC (**G**, **H**). NAC treatment is able to improve microphthalmia (vertical dashed lines in **G** compared to **F**) and microcephaly (horizontal dashed lines in **G** compared to **F**) in 60% of morphant embryos (**H**). Scale bars: 100 µm.

When the respiratory chain is inhibited downstream of complex III, electrons coming from succinate oxidation can lead to ROS generation by reverse electron transport from complex II to complex I (Lambert & Brand, 2004; St-Pierre et al, 2002). ROS production can also increase when electron transport is reduced as a consequence of low respiratory rates (Korshunov et al, 1997) or in pathological situations associated to MRC defects (Wallace, 2005). Accordingly, H_2_O_2_-induced growth inhibition was markedly increased in the B-8025-Δ*cyc3* strain defective for heme lyase function (Supporting Information Fig S8), as were ROS levels in *hccs*-morphants as determined by accumulation of oxidized CM-H_2_DCFDA (Fig 6A). Notably, *in vitro* experimental evidence suggested that ROS could directly mediate mitochondrial caspase-9 auto-activation, inducing cell death via an apoptosome-independent pathway (Katoh et al, 2008, 2004). We thus hypothesized that caspase-dependent cell death induced by hccs deficiency could be linked to MRC impairment and ROS overproduction. Indeed, detection of mitochondrial superoxide by MitoSOX staining revealed a specific increase of mitochondrial ROS levels in the CNS of alive morphants compared to controls (Supporting Information Fig S9).

We thus tested whether mitochondrial ROS overproduction is responsible for caspase activation by treating *hccs*-morphants with *N*-acetyl-cysteine (NAC), a ROS scavenger compound. Notably, NAC treatment strongly reduced cell death in morphants (Fig 6B–D) and counteracted the increased level of caspase-9 activation observed in the mitochondrial fractions from morphant embryos, as compared to that of controls (Fig 6E), finally rescuing both microphthalmia and microcephaly (Fig 6F–H).

Altogether, these results demonstrate that increased ROS production due to MRC impairment induce caspase-9 activation in the mitochondria thus triggering tissue-specific PCD, ultimately leading to the MLS phenotype.

## DISCUSSION

Apoptosis of neuronal precursors and of differentiating neurons is a key process in CNS development. This process needs to be accurately regulated and coordinated to ensure the generation of the proper number of terminally differentiated cells for each of the many neuronal cell types that compose the mature CNS (Iijima & Yokoyama, 2007; Valenciano et al, 2009; Vecino et al, 2004). Deregulation of this process results in altered neuronal circuitries and underlies different human pathological conditions (Hotchkiss et al, 2009; Mattson et al, 2008; Ribe et al, 2008; Wang et al, 2009). In this context, fine regulation of the canonical mitochondrial-dependent cell death pathway seems to be crucial for the correct development of the eye (Guerin et al, 2006; Isenmann et al, 2003; Laguna et al, 2008).

Here we demonstrate that activation of a non-canonical mitochondria-dependent cell death pathway underlies microphthalmia and microcephaly in the animal model for MLS syndrome we have generated. MLS is a rare syndromic form of microphthalmia caused by mutation in *HCCS*, a nuclear gene codifying for a key player for mitochondrial respiration. Our results clearly showed that the mitochondrial protein HCCS plays a key role during eye and brain development through the control of mitochondrial PCD. On the basis of our results, we propose that MLS should be classified as a mitochondrial disease even if its salient features differ from those found in ‘canonical’ mitochondrial disorders, which are usually characterized by postnatal failure rather than impaired organogenesis.

HCCS catalyses covalent attachment of heme moieties to both apo-Cytc and c_1_. By combining *in vivo* experiments and functional approaches in yeast, we first showed that HCCS is required to maintain the activity of the MRC complexes III–IV and directly controls the import of Cytc into mitochondria independently from its heme lyase activity. This clearly demonstrates that HCCS binding to apo-Cytc, rather than heme attachment, is the critical step for Cytc import into mitochondria. This possibility had been previously suggested (Dumont et al, 1991) although, to date, convincing experimental evidence was not available. Moreover our results indicate that the *HCCS* gene mutations found in MLS patients exert their pathogenic effect via OXPHOS impairment.

**Figure 7:**
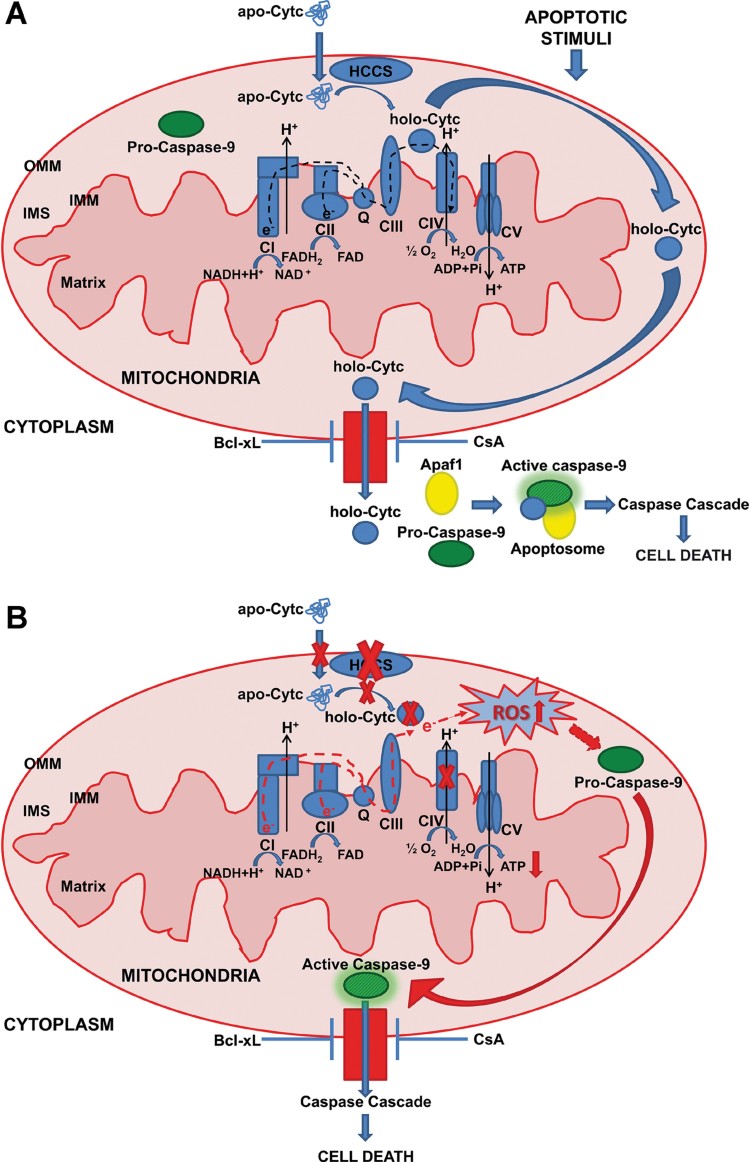
Model for apoptosome-independent, ROS-dependent, mitochondrial caspase-9 activation in the absence of HCCS The mitochondrial electron transfer system, complexes I–V (CI-CV), is illustrated. Red arrows indicate an increase (upward) or decrease (downward) in each physiological parameter. Bar-headed lines indicate inhibition. OMM, outer mitochondrial membrane; IMS, intermembrane space; IMM, inner mitochondrial membrane. Canonical mitochondrial-dependent cell death pathway. In the presence of HCCS, upon apoptotic stimuli, holo-Cytc is released from mitochondria and binds Apaf1 and Pro-caspase-9 in the cytoplasm inducing the formation of the apoptosome and the sequential activation of caspase-9.In HCCS deficient condition the lack of holo-Cytc leads to inhibition of the MRC with the subsequent increase of ROS levels, which results in Apaf1-independent caspase-9 activation into mitochondria.

Besides their role in energy production, mitochondria are also key regulators of PCD through the mitochondrial-dependent pathway. According to previous studies, in the absence of hccs, cells should not activate the canonical intrinsic apoptotic pathway owing to the absence of Cytc (Li et al, 2000; Fig 7A). However, our results provide the experimental evidence that hccs deficiency induces a non-canonical caspase-9 activation that is independent from apoptosome formation and occur into mitochondria. We hypothesized that hccs deficiency might induce caspase activation and cell death by MRC impairment and overproduction of ROS, mostly derived from complex III (St-Pierre et al, 2002). Interestingly, antioxidant treatment significantly ameliorated the phenotype of *hccs*-morphants indicating that enhanced levels of ROS due to MRC impairment triggers PCD, ultimately leading to the CNS abnormalities observed in morphants. Therefore, we conclude that hccs, besides driving Cytc synthesis, is endowed with a previously unknown role in a novel start-up of non-canonical cell death pathway (Fig 7B). The correct regulation of this pathway can be crucial for the proper development of the brain and the eyes. Our data strongly support a role for mitochondrial caspase-9 activation in pathological conditions. ROS seem to act as cell signalling molecules with a central role in physiological cellular responses (Miki & Funato, 2012). Therefore, the non-canonical cell death pathway we described here may likely operate in different physiological context that we hope to elucidate in the future.

Although *HCCS* is ubiquitously expressed and is required for cellular respiratory function, the phenotypic traits observed in MLS patients are restricted to specific organs, suggesting that dosage and function of HCCS are critical in selected tissues. Accordingly, *hccs*-morphants presented a dramatic increase in caspase-dependent apoptosis in the eye and in other CNS regions, but not in the heart. The latter observation is in line with the phenotype observed after conditional inactivation of *Hccs* in the murine heart, in which no apoptotic cardiomyocytes could be detected (Drenckhahn et al, 2008). Similarly, in a zebrafish model of OXPHOS deficiency, the knockdown of a subunit of complex IV increased apoptotic cell death but only in the CNS (Baden et al, 2007). A possible explanation is the differential tissue sensitivity to mitochondrial ATP depletion (high *vs.* low energy demand) and/or ROS overproduction as also proposed to explain the phenotypic complexity of other OXPHOS deficiencies (Floreani et al, 2005; Rossignol et al, 1999; Wallace, 2005). For example, impairment of the respiratory chain and/or enhanced ROS production may elicit different molecular responses in selected organ/tissue types depending on their endogenous antioxidant levels (Wallace, 2005). Furthermore, requirement and coupling of caspase-9 with Apaf1 and Cytc could be both context-dependent and cell-type specific (Hao et al, 2005; Ho et al, 2004; Katoh et al, 2008). The molecular mechanisms by which hccs regulates caspase-9 activation in the mitochondria deserves more extensive investigation. These studies will help explaining why specific tissues and organs are preferentially affected in MLS syndrome. However, the evidence of an increased apoptosis in the CNS combined with the reduced proliferation of Hccs-defective cardiomyocytes (Drenckhahn et al, 2008), suggest that the phenotype observed in MLS syndrome may result from the tissue specific activation of different molecular networks. This hypothesis may account for the specific clinical signs observed in the disease, whereas the influence of X-inactivation remains the best explanation for the high degree of intra-familiar clinical variability observed in MLS patients (Morleo & Franco, 2008; Van den Veyver, 2001).

Mitochondrial PCD is a key step for proper eye development (Guerin et al, 2006; Isenmann et al, 2003; Laguna et al, 2008), and we have now demonstrated that HCCS has a crucial role in this process. To the best of our knowledge, HCCS and COX7B, that has been recently implicated in the MLS syndrome (Indrieri et al, 2012), are the first examples of MRC-related proteins involved in the pathogenesis of a hereditary form of microphthalmia. The molecular basis of many inherited developmental disorders involving the CNS still remains to be determined. On the basis of our results, mitochondrial-related genes should be considered as possible candidates for these conditions.

In summary, our combined approaches demonstrated that HCCS controls a novel start-up of a non-canonical cell death pathway in the brain and the eyes, via enhanced mitochondrial ROS production and release of the active caspase-9 from the mitochondria. Our work also shows that this apoptotic cascade is responsible for the microphthalmia and the other CNS defects present in the MLS syndrome. Altogether, our data provide experimental evidence for a mechanistic link between intrinsic apoptosis, mitochondrial diseases and developmental disorders.

## MATERIALS AND METHODS

### Yeast complementation studies

We used *S. cerevisiae* strain B-8025 (MATα *can1-100 CYC1 cyc3-*Δ*cyc7-*Δ*::CYH2 cyh2 his3-*Δ*1 leu2-3,112 trp1-289*; B-8025-Δ*cyc3*), which carries a deletion of *CYC3*, as well as B-7553 (MATα *can1-100 CYC1 cyc7::CYH2 cyh2 his3-*Δ*1 leu2-3,112 trp1-289*) as control (Wimplinger et al, 2006). For complementation studies B-8025-Δ*cyc3* was transformed with pYEX-HCCS, pYEX-R217C, pYEX-Δ197-268, pYEX-E159K and the pYEX empty vector as control (Wimplinger et al, 2006, 2007). Cells were cultured in yeast nitrogen base (YNB) medium [0.67% YNB without amino acids (ForMedium™, Hunstanton, UK)] supplemented with 1 g/L of drop-out powder containing all amino acids except those required for plasmid maintenance. Various carbon sources were added at 2% w/v (Carlo Erba Reagents, Milan, Italy). Media were solidified with 20 g/L agar (ForMedium™). For the cytochromes absorption spectra, respiration and mitochondria extraction cells were grown to late-log phase in the YNB medium supplemented with 0.6% glucose. Cells were cultured and plates were incubated at 33°C.

### Cytochrome spectra and respiration

Differential spectra between reduced and oxidized cells of a suspension of cells at 60 mg/ml (wet weight) were recorded at room temperature, using a Cary 300 Scan spectrophotometer (Varian, Palo Alto, CA, USA). Oxygen uptake rate was measured at 30°C using a Clark electrode in a reaction vessel of 3 ml of air-saturated respiration buffer (0.1 M phthalate–NaOH, pH 5.0), 10 mM glucose, starting the reaction with the addition of 10 mg of wet weight of cells as previously described (Ferrero et al, 1981).

### Isolation of yeast mitochondria and Western blot analysis

Preparation of yeast mitochondria was performed as previously described (Glick & Pon, 1995). Protein concentration was determined using the Bio-Rad Protein Assay (Bio-Rad, München, Germany) following the manufacturer's instructions. The detection of Cytc content into mitochondria was performed by Western blot (WB) analysis. A total of 40 µg mitochondrial protein/lane were loaded on 12% SDS–polyacrylamide gel. For WB analysis, the gels were electroblotted onto nitrocellulose filters and sequentially immunostained with polyclonal antibody against Cytc (1:1000; gift from Dr. Antonio Barrientos), and Atp4 (ATP synthase, subunit 4; 1:10,000; gift from Dr. Jean Velours). A peroxidase-conjugated anti-rabbit was used at 1:10,000 as secondary antibody (GE Healthcare, Little Chalfont Buckinghamshire, UK).

### Medaka stocks

Wild-type *O. latipes* of the cab strain were maintained in an in-house facility (28°C on a 14/10 h light/dark cycle). Embryos were staged as described (Iwamatsu, 2004).

### Ethics statement

All studies on fishes were conducted in strict accordance with the institutional guidelines for animal research and approved by the Italian and Spanish Ministry of Health; Department of Public Health, Animal Health, Nutrition and Food Safety in accordance to the law on animal experimentation (article 7; D.L. 116/92; protocol number: 00001/08/IGB; approval date October 22, 2008). Furthermore, all animal treatments were reviewed and approved in advance by the Ethics Committee of the Institute of Genetics and Biophysics, IGB Animal House (Naples, Italy) and of the CSIC.

### MOs and mRNAs injections

MOs (Gene Tools, LLC Philomath, OR, USA) were designed and injected into fertilized embryos at the one/two-cell stage. cDNA sequences corresponding to *Bcl-xL*, green fluorescent protein (GFP), red fluorescent protein (RFP) as well as the mutated version of *hccs* resistant to its own MO were cloned into the pCS2^+^ vector and the corresponding mRNAs were transcribed using the SP6 mMessage mMachine kit (Ambion, Inc., Austin, TX, USA) according to manufacturer's instructions. Rescue experiments were performed by co-injecting *hccs* or *Bcl-xL* mRNA with the *hccs*-MO into one blastomere of the embryos at the one/two cell stage. eGFP mRNA was always included in the injection solutions as reporter. MO sequences, their working concentrations and mRNA and MO injection conditions are described in Supporting Information Materials and Methods section and Supporting Information Table S1. The specificity and efficiency of MO were determined as described (Eisen & Smith, 2008; see Supporting Information Materials and Methods section and Supporting Information Fig S3). At least three independent experiments were performed for each marker and condition.

### Whole-mount ISH

Whole-mount RNA ISH was performed, sectioned and photographed as reported (Conte et al, 2010). The cDNA of the *hccs* gene was isolated by RT-PCR amplification with the specific primers listed in Supporting Information Materials and Methods section.

### Isolation of mitochondria and Western blot analysis in medaka

Crude mitochondrial and cytoplasmic fractions from 80 to 100 st30 embryos were isolated using a MitoIso 1 isolation kit (Sigma–Aldrich, St. Louis, MO, USA) as previously described (Anichtchik et al, 2008) and the protein concentration was assayed using the Bio-Rad Protein Assay (Bio-Rad, München, Germany). For Western blotting analysis, the gels were electroblotted onto nitrocellulose filters and sequentially immunostained with anti-Cytc (1:500; BD Pharmingen™, San Diego, CA, USA), anti-COXIV (cytochrome c oxidase, subunit 4; 1:1000; Cell Signaling Technology, Danvers, MA, USA) and anti-GAPDH (1:1000; Santa Cruz Biotechnology, Santa Cruz, CA, USA) antibodies. Peroxidase-conjugated anti-rabbit and anti-mouse antibodies were used at 1:3000 as secondary antibody (GE Healthcare).

**The paper explained**

PROBLEM:

Mitochondrial-dependent programmed cell death is very important in normal tissue/organ development. However, the link between mitochondrial dysfunction and developmental disorders has not yet been determined.

RESULTS:

We demonstrate that inactivation of holo-cytochrome c-type synthase (*HCCS*), a transcript important for the mitochondrial respiratory chain (MRC), is associated with unconventional activation of caspase-9 in the mitochondria triggered by MRC impairment and overproduction of reactive oxygen species. We also show that this mechanism can explain the phenotype observed in a developmental disorder, microphthalmia with linear skin lesions syndrome (MLS) associated with mutations in the *HCCS* transcript.

IMPACT:

Our results provide the first experimental evidence for a mechanistic link between mitochondrial dysfunction, intrinsic apoptosis, and developmental disorders. Mutations in other MRC-related genes may well underlie severe developmental disorders hallmarked by a respiratory phenotype and caused by activation of mitochondrial apoptosis.

### Immunolabelling and TUNEL assay

Medaka embryos were cryostat-sectioned, and immunochemistry was performed as described (Flynt et al, 2007) using anti-phospho-histone H3 monoclonal antibody (1:100; Cell Signaling Technology), α-active-caspase-3 (1:500; R&D System, Minneapolis, MN, USA). Alexa-488–conjugated goat anti-rabbit (1:1,000; Invitrogen, Eugene, OR, USA) IgGs were used as secondary antibodies. Alternatively, a peroxidase-conjugated anti-rabbit antibody (1:200; Vector Laboratories, Burlingame, CA, USA) was used followed by diaminobenzidine staining, as described (Conte et al, 2010). TUNEL assays were performed using In Situ Cell Death Detection Kit (Roche, Mannheim, Germany) according to manufacturer's instructions.

### Transmission electron microscopy (TEM)

The embryos were fixed overnight at 4°C in 2.5% glutaraldehyde/2% paraformaldehyde in PBS. Then, they were rinsed in PBS buffer and post-fixed in 1% osmium tetroxide for 1 h at 4°C. After dehydration in a graded series of ethanol, the embryo were embedded in an Epon 812 resin (Polyscience, Niles, IL, USA). Ultrathin sections (60–90 nm) were cut on a Leica Ultracut UCT ultramicrotome (Leica Microsystems, Mannheim, Germany) and contrasted with uranyl acetate and lead citrate. Grids were examined using a transmission electron microscope JEM-1011 (JEOL, Tokyo, Japan) at 100 kV and micrographs were taken with the iTEM software (Olympus Soft Imaging System GmbH, Münster, Germany).

### Caspase assays

Caspase assays were performed as previously reported (Stanton et al, 2006) on total lysates and mitochondrial and cytoplasmic fractions according to manufacturer's instructions. For the assay on total lysates 20 embryos for sample were dechorionated at st30 and quick frozen in liquid nitrogen for at least 24 h. The embryos were then thawed, suspended in 100 µl of lysis buffer (20 mM Hepes-KOH, pH 7.5, 250 mM saccharose, 50 mM KCl, 2.5 mM MgCl_2_ and 1 mM dithiothreitol) and passed through a 26.5-gauge needle. The embryo lysate was incubated on ice for 30 min and then centrifuged at 10,000 rpm for 15 min at 4°C. One hundred microlitres of the caspase-3/7-, caspase-8-, and caspase-9-glo luciferase reagent (Promega, Madison, WI, USA) was added in a 1:1 ratio with 10 µl of the supernatant diluted with 90 µl lysis buffer and incubated at room temperature in the dark for 2 h.

For the assay on mitochondrial and cytoplasmic fractions 100 µl of the caspase-9-glo luciferase reagent was added in a 1:1 ratio with 10 µl of the supernatant diluted with 90 µl mitochondrial storage buffer from MitoIso 1 isolation kit (Sigma–Aldrich) and incubated at room temperature in the dark for 30 min.

For both assays the emitted luminescence signals were normalized for the protein concentration of each sample.

### Caspase inhibitors

The following caspase inhibitors (CalBiochem, La Jolla, CA, USA) were used: Z-VAD-FMK as a general inhibitor for caspases, Ac-YVAD-CHO for caspase-1 and caspase-4, and Ac-LEHD-CMK for caspase-9. Stocks were diluted in DMSO or distilled water at a final concentration of 5 µM. Caspase inhibitors were injected into fertilized embryos at the one/two cell stage as previously described (Walker & Harland, 2009).

### Detection of ROS levels

ROS accumulation, assessed by the levels of the oxidized form of the cell-permeant ROS indicator acetyl ester of 5-(and 6-) chloromethyl-2′,7′-dichlorodihydrofluorescein diacetate (CM-H_2_DCFDA; Invitrogen), was detected in living embryos at st24 as described (Anichtchik et al, 2008). *In vivo* mitochondrial levels of superoxide were detected using MitoSOX (Invitrogen). Dechorionated living embryos at st24 were incubated with 0.5 µM MitoSOX dye for 20 min in the dark, rinsed thoroughly, mounted in low melt agarose and analysed using LSM 700 Zeiss confocal microscope (Carl Zeiss International, Germany). Images were captured using the ZEN software (Carl Zeiss International). Z-stack views were taken and combined in one single image.

### *N*-acetyl-cysteine (NAC) and cyclosporine (CsA) treatments

Embryos were dechorionated at gastrula stage and incubated with 100 µM NAC dissolved in the embryo medium. For CsA treatments morphant embryos were dechorionated at st30 and then incubated in 100 µM CsA for 2 h. CsA was dissolved in 0.1% DMSO in the embryo medium. Vehicle control treatment consisted of 0.1% DMSO in embryo medium.

### Statistical analysis

In all experiments the significance of differences between groups was evaluated by ANOVA or by Student's *t*-test, *p* < 0.05 was considered significant. Quantitative data are presented as the mean ± SD (standard deviation) or ±SEM (standard error of the mean) of at least three independent experiments.

## For more information

OMIM, Online Mendelian Inheritance in Man, MLS syndrome http://omim.org/entry/309801

Portal for rare diseases and orphan drugs http://www.orpha.net/

Ensembl medaka genome http://www.ensembl.org/Oryzias_latipes/Info/Index

Telethon Institute of Genetics and Medicine, http://www.tigem.it/en

## Supplementary material

As a service to our authors and readers, this journal provides supporting information supplied by the authors. Such materials are peer reviewed and may be re-organized for online delivery, but are not copy-edited or typeset. Technical support issues arising from supporting information (other than missing files) should be addressed to the authors.
