# The impairment of HCCS leads to MLS syndrome by activating a non-canonical cell death pathway in the brain and eyes

**DOI:** 10.15252/emmm.201470060

**Published:** 2014-06-03

**Authors:** Alessia Indrieri, Ivan Conte, Giancarlo Chesi, Alessia Romano, Jade Quartararo, Rosarita Tatè, Daniele Ghezzi, Massimo Zeviani, Paola Goffrini, Ileana Ferrero, Paola Bovolenta, Brunella Franco

**Correction to:** *EMBO Mol Med* (2013) 5: 280–293. DOI 10.1002/emmm.201201739

In the above article, holo-cytochrome c-type synthase was used instead of holocytochrome c-type synthase, the official gene name. This error occurs in three places in the text and the correct sentences should read:

In the abstract:

Now we provide the evidence that non-canonical mitochondrial-dependent apoptosis explains the phenotype of microphthalmia with linear skin lesions (MLS), an X-linked developmental disorder caused by mutations in the holocytochrome c-type synthase (HCCS) gene.

In the introduction:

HCCS is a highly conserved gene from fungi to metazoans and encodes a mitochondrial holocytochrome c (Cytc)-type synthase, also known as ‘heme lyase’, located on the outer surface of the inner mitochondrial membrane (Schaefer *et al*, 1996; Schwarz & Cox, 2002).

In ‘The paper explained’ section:

We demonstrate that inactivation of holocytochrome c-type synthase (HCCS), a transcript important for the mitochondrial respiratory chain (MRC), is associated with unconventional activation of caspase-9 in the mitochondria triggered by MRC impairment and overproduction of reactive oxygen species.

