# Supplementary material for: The impairment of HCCS leads to MLS syndrome by activating a non-canonical cell death pathway in the brain and eyes
Source: EMBO Mol Med. 2013 Jan 22;5(2):280–93. doi: 10.1002/emmm.201201739 (PMC3569643; doi:10.1002/emmm.201201739)
Supplement: Supplementary file 1 [file emmm0005-0280-SD1.pdf]

EMBO Molecular Medicine

The impairment of HCCS leads to MLS syndrome by activating a non-canonical cell death pathway in the brain and eyes

Alessia Indrieri, Ivan Conte, Giancarlo Chesi, Alessia Romano, Jade Quartararo, Tatè Rosarita, Daniele Ghezzi, Massimo Zeviani, Paola Goffrini, Ileana Ferrero, Paola Bovolenta, Brunella Franco

Corresponding author: Brunella Franco, TIGEM

Review timeline:

Submission date:	13 July 2012
Editorial Decision:	15 August 2012
Revision received:	15 November 2012
Editorial Decision:	02 December 2012
Revision received:	03 December 2012
Accepted:	04 December 2012

Transaction Report:

(Note: With the exception of the correction of typographical or spelling errors that could be a source of ambiguity, letters and reports are not edited. The original formatting of letters and referee reports may not be reflected in this compilation.)

1st Editorial Decision 15 August 2012

Thank you for the submission of your manuscript "HCCS, the Gene Responsible for MLS Syndrome, Regulates Activation of a Non-Canonical Cell Death Pathway in brain and eye explaining the disease phenotype" to EMBO Molecular Medicine. We have now heard back from the three referees whom we asked to evaluate your manuscript. You will see that they find the topic of your manuscript potentially interesting. However, they also raise significant concerns on the study, which should be addressed in a major revision of the manuscript.

In particular, reviewer #3 highlights that further experimental evidence to support proposed caspase-9 activation in mitochondria should be provided. In addition, reviewer #1 notes that the evidence for mitochondrial dysfunction after HCCs knockdown should be strengthened. Of note, reviewer #1 and #3 mention that the yeast studies are not central to the manuscript and thus suggest to move them to the Supporting Information section.

On a more editorial note, the description of all reported data that includes statistical testing must state the name of the statistical test used to generate error bars and P values, the number (n) of independent experiments underlying each data point (not replicate measures of one sample), and the actual P value for each test (not merely 'significant' or 'P < 0.05'). In addition, for primary research manuscripts in EMBO Molecular Medicine that report experiments on live vertebrates and/or higher invertebrates, the corresponding author must confirm that all experiments were performed in accordance with relevant guidelines and regulations. The manuscript must include in the Methods section, Supporting Information or, if brief, at an appropriate place within the text of article, a statement identifying the institutional and/or licensing committee approving the experiments, including any relevant details. Please see our Guide to Authors for more information

Given the balance of these evaluations, we feel that we can consider a revision of your manuscript if you can convincingly address the issues that have been raised within the space and time constraints outlined below.

Revised manuscripts should be submitted within three months of a request for revision. They will otherwise be treated as new submissions, unless arranged otherwise with the editor.

I look forward to seeing a revised form of your manuscript as soon as possible.

Yours sincerely,

Scientific Editor
EMBO Molecular Medicine

***** Reviewer's comments *****

Referee #1:

Mutations in holo-cytochrome c synthase (HCCS) cause the MLS syndrome, which is characterized by remarkable tissue specificity. Here the authors establish a medaka model for this disease and demonstrate that morpholino knockdown of HCCs phenocopies hallmarks of the disease. Loss of HCCS is shown to trigger non-canonical, apoptosome-independent apoptosis, which can be blocked by caspase 9 inhibitors, CsA, Bcl2 overexpression or treatment with NAC, indicating that it involves ROS and caspase 9-release from the intermembrane space of mitochondria. Indeed, the knockdown of HCCS impairs complex III activity moderately and leads to an increased ROS, which could activate caspase 9.

This is an interesting and important study on a novel model for MLS, which provides convincing evidence for the activation of non-canonical apoptotic pathway in the absence of HCCS as a possible pathogenic mechanism in MLS. However, the authors should consider the following points:

1. The dysfunction of mitochondria upon HCCS knockdown is not convincingly documented. The decrease in complex III activity is rather moderate and other RC complexes have not been analyzed. Statistics for appearance of mitochondria with an aberrant morphology were not provided. The authors should also enlarge Fig. 3G/H¥
2. The number of normal hccs-MO embryos is increased upon NAC treatment suggesting deleterious effects of an increased ROS production. Does NAC treatment suppress the activation of caspase 9 (as monitored in Fig. 6H)?
3. Is it possible to assess cytochrome c levels in hccs-MO?
4. The experiments in yeast (Fig. 1) appear rather disconnected from the rest of the manuscript which focusses on the medaka model. The well-performed experiments demonstrate functional conservation but otherwise are of limited novelty and therefore may be deleted from the manuscript.

Referee #2 (Comments on Novelty/Model System):

The technical quality of the experimental setting is high and the medaka fish is an adequate genetic model system.

Referee #2 (Other Remarks):

EMM-2012-01739 "HCCS, the Gene Responsible for MLS Syndrome, Regulates Activation of a

Non-Canonical Cell Death Pathway in brain and eye explaining the disease phenotype" by Dr. A. Indrieri et al.

COMMENTS TO THE AUTHORS

In this paper, Indrieri and colleagues examine a medaka model which recapitulates the phenotype of Microphthalmia with Linear Skin lesions (MLS), an X-linked developmental disorder caused by mutations in the Holo-Cytochrome c-type synthase (HCCS) gene. The study shows that downregulation of hcc causes increased cell death via an apoptosome-independent caspase-9 activation in brain and eyes. Thereby, caspase-9 activation in the mitochondria is triggered by impairment of the mitochondrial respiratory chain and an overproduction of reactive oxygen species. This is a useful study which indicates that HCCS is a key molecule in the development of the central nervous system that modulates a new non-canonical cell death and provides the first experimental evidence for a mechanistic link between mitochondrial dysfunction, intrinsic apoptosis and developmental disorders.
There are, however, some major and minor comments the authors may wish to consider.

Major criticisms:

1. The authors propose that increased ROS production, caused by an impairment of the mitochondrial respiratory chain triggers tissue-specific programmed cell death, leading to MLS. It would be of interest, which ROS species are increased. Please specify.
2. The yeast studies are very interesting; please confirm the results for cytochrome C expression in the medaka model (western blot analysis of cytosolic and mitochondrial fraction).

Minor criticisms:

Page 1, keywords: change to "... Holo-Cytochrome c-type synthase ..."
Page 3, line 9: Morleo & Franco 2009 is missing in the "References"
Page 6, line 9: change to "... MRC complexes ..."
Page 8, line 26: change to "... (10 {plus minus} 5%) ..."
Page 10, line 1: change to "... XIAP ..."
Page 10, line 4: change to "... apoptosome complex ..."
Page 10, line 19-20: change to "... caspase-8 ... caspase-9 ..."
Page 13, line 12: change to "...Holo-Cytochrome c-type synthase ..."
Page 15, line 1: change to "... complexity..."
Page 17, line 7: "...ForMedium{trade mark, serif}, UK ..." City?
Page 17, line 9: "...Carlo Erba Reagents, Italy ..." City?
Page 17, line 9: change to "... 20 g/l..."
Page 17, line 12: change to "... 33 {degree sign}C..."
Page 17, line 14: "... Cary 300 Scan spectrometer..." Company, City, Country?
Page 17, line 15: change to "... 30 {degree sign}C... 3 ml..."
Page 17, line 16: change to "... 0.1 M...10 mM..."
Page 17, last line: "... Bio-Rad Protein Assay..." Company, City, Country?
Page 18, line 4: "... Cytc, Atp4..." Company, City, Country?
Page 18, line 8: change to "... 28 {degree sign}C..."
Page 18, line 11: "... (Gene Tools, LLC)..." Company, City, Country?
Page 18, line 15: "... (Ambion)..." City, Country?
Page 19, line 5: change to "... and TUNEL assay ..."
Page 19: "... (Cell Signaling Technology)... (BD Pharmingen{trade mark, serif}) ... (Vector laboratories) ... (Roche) ... (Leica Microsystems) ..." City, Country?
Page 19, line 14, 15: change to "... 4 {degree sign}C..."
Page 19, line 16: change to "... Epon 812 ..."
Page 19, last line: change to "... Olympus Soft Imaging System GmbH, M¸nster, ..."
Page 20, line 1: change to "... Caspase Assays ..."
Page 20, line 5: change to "... 50 mM ... 2.5 M MgCl2"
Page 20, line 7: change to "... 100 µl ..."
Page 20, line 8: "... (Promega)..." City, Country?
Page 20, line 9: change to "... 2 h ..."

Page 20, last line: change to "... Olympus Soft Imaging System GmbH, M¸nster, ..."
Page 20, line 13: Ac-YVAD-CHO is a caspase-1 and caspase-4 inhibitor!
Page 21, line 1: change to "... 2 h ..."
Page 28, line 8: change to "... Western blot ..."
Figure 1B: Unit of the "Respiratory activity" is missing!
Figure 8: change "CYTOPLAMS" to "CYTOPLASM"
SUPPORTING INFORMATION Page 2, line 1: change to "... 10-400 µM ..."
SUPPORTING INFORMATION Page 2, line 3: change to "...100 ng ... 25 ng ..."
SUPPORTING INFORMATION Page 2, last line: change to "... 300 µM ..."
SUPPORTING INFORMATION Page 3, line 1: change to "... 400 µM ..." Remove the red highlights!
SUPPORTING INFORMATION Page 3, line 2: change to "... 30 µM ..."
SUPPORTING INFORMATION Page 3, line 3, 4: change to "... 100 µM ..."
SUPPORTING INFORMATION Page 3, line 10: change to "... 33 {degree sign}C ..."
SUPPORTING INFORMATION Page 3, line 20: change to "... -80 {degree sign}C ..."
SUPPORTING INFORMATION Page 3, line 21: change to "... 250 mM ... 20 mM ... 3 mM ..."
Supporting Table S1: Stated are standard deviations (SD), but in the "Materials and Methods" (Page 20) "Quantitative data are presented as the mean {plus minus} SEM" is denoted. Please explain.
Supporting Figure S2, Figure H: SEM is missing!
Supporting Figure S2, line 2: change to "... st38 ..."
Supporting Figure S2, line 8: change to "... st19 ..."

Referee #3 (Comments on Novelty/Model System):

This study provides convincing data about the pathological cause of the microphtalmia in MLS syndrome, an issue that has not been address before because of the lack of a good model for the disease. Although MLS syndrome is a rare disease, the study has a broader interest as it provides insights into the current knowledge of caspase-9 mediated cell death.
The study is very good and it fits to the scope of the journal. Experiments are well thought and, in general, nicely executed.
Although the study does not address the mechanistic link between increased ROS and caspase-9 activation its contribution to the understanding of the disease is significant. In my opinion a revised version of the paper addressing the comments and suggestions made should be sufficient for acceptance of the paper for publication. Suggested additional experiments to support activation of mitochondrial caspase-9 as the underlying cause of the disease are desirable but not a requisite for publication of the paper.

Referee #3 (Other Remarks):

This study focuses on MLS syndrome, an X-linked disease characterized by unilateral or bilateral microphtalmia and linear skin defects, which is caused by mutations in HCCS. This gene encodes a mitochondrial enzyme required for the maturation of Cytc and Cytc1. To model the disease the authors used morpholinos and knocked-down the hccs gene in the medaka fish. They performed a set of experiments and show that microphtalmia caused by dowregulation of the medaka hccs gene is the consequence of an increased developmental cell death that involves activation of caspase-9 and caspase-3/7. They also show that caspase-9-mediated cell death in the eyes of Hccs knockdown embryos does no require Apaf-1 and provide evidences indicating that activation of caspase-9 occurs within the mitochondria. They go on and show that production of ROS in Hccs knockdown embryos is higher than in controls. Base on these experiments, and on experiment performed in yeast strains that express human HCCS mutant alleles, they conclude that microphtalmia in MLS syndrome is due to the activation of a non-canonical mitochondrial-dependent cell death pathway.

Results present in this manuscript are novel and provide a mechanistic explanation for microphtalmia in MLS syndrome. They also provide new insights into the current knowledge of caspase-9 mediated cell death that are of broader interest. The authors should address the following

comments and questions before acceptance of the paper for publication.

Comments and questions:

1. One of the important findings of the study is the activation of caspase-9 by a mechanism that does not require the formation of the apoptosome. Based on the experiments shown in Figure 6 (D to H), the authors propose a model by which activation of caspase-9 occurs within the mitochondria. Further experimental evidences (i.e.: measurements of caspase-9 activation in mitochondrial and cytoplasmic fractions) would strengthen the proposed model.

2. The authors cite examples of apoptosome-independent caspase-9 activation (page 11). They should also include the work by Manns and colleagues published last year in FASEB J.

3. What is the contribution of mitochondrial caspase-9 activation (Apaf1-independent) in normal developmental cell death? In other words, is this non-canonical cell death pathway relevant in physiological conditions or is activated only in situations of a pathological increase of ROS? This issue should be addressed in the discussion section and the Title of the manuscript and last sentence of the Abstract modified accordingly.

4. Published data about developmental apoptosis in the Medaka nervous system (i.e. Iijima and Yokoyama; Acta Histochem. Cytochem. 40 (1): 1-7, 2007) should be cited in the manuscript and taking into account for presentation and discussion of the results.

5. Results in yeast presented in Figure 1 are not fundamental for the conclusion of the paper. This data could be moved to Supplemental information or after the results presented in Figure 5.

6. Conclusions of the results shown in Figures 1A and C are not clear (page 7). A description of the human HCCS mutations would facilitate interpretation of the data.

7. Developmental cell death is one of the mechanisms, but not the most common mechanism, that regulates the size of a cell population. First sentence, second paragraph, in page 4 is not correct and needs to be written again.

8. To avoid unnecessary repetitions, the paragraph describing the canonical intrinsic cell death pathway in the Introduction section (pages 4-5) should be summarized or eliminated from the Introduction section.

9. Cell death in the central nervous system affects neuroepithelial progenitors as well as differentiating neurons (reviewed in Valenciano et al, 2009). First paragraph in the Discussion section needs to be written accordingly to this revision.

10. The eye phenotype in st24 embryos injected with Hccs-MO seems more severe than in st30 (Figures 4 C and D). Is the section shown in Figure 4C representative of the phenotype?

Minor issues:

- Text in histograms shown in Figures 4, 5, 6 and 7 are difficult to read.
- Figure 8 needs some modifications; schemes are not clear and letters too small.

1st Revision - authors' response 15 November 2012

Point by point response to reviewers

Referee #1:

Mutations in holo-cytochrome c synthase (HCCS) cause the MLS syndrome, which is characterized by remarkable tissue specificity. Here the authors establish a medaka model for this disease and demonstrate that morpholino knockdown of HCCs phenocopies hallmarks of the disease. Loss of HCCS is shown to trigger non-canonical, apoptosome-independent apoptosis, which can be blocked by caspase 9 inhibitors, CsA, Bcl2 overexpression or treatment with NAC, indicating that it involves ROS and caspase 9-release from the intermembrane space of mitochondria. Indeed, the knockdown of HCCS impairs complex III activity moderately and leads to an increased ROS, which could activate caspase 9.
This is an interesting and important study on a novel model for MLS, which provides convincing evidence for the activation of non-canonical apoptotic pathway in the absence of HCCS as a possible pathogenic mechanism in MLS. However, the authors should consider the following points:

1. The dysfunction of mitochondria upon HCCS knockdown is not convincingly documented. The decrease in complex III activity is rather moderate and other RC complexes have not been analyzed. Statistics for appearance of mitochondria with an aberrant morphology were not provided. The authors should also enlarge Fig. 3G/H';

We appreciate the concern, however we would like to stress that there are a number of technical and conceptual reasons that explain the observed moderate decrease in complex III activity. a) Morpholinos normally knock-down the level of gene expression but rarely abolish it completely (Eisen and Smith, Development 2008). This has been a critical advantage in our case, because this characteristic allowed us to obtain a viable model for this embryonic lethal disease. As a consequence, we expect a certain degree of activity of the enzyme. b) In order to perform the assay we need to pool enough st19-24 embryos to obtain the necessary amount of protein extract. The majority of hccs morphants with high level of hccs inactivation will not survive up to this stage, probably because low levels of hccs are not compatible with life. Indeed, genetic inactivation of hccs results in ES cell lethality in the mouse

(Prakash et al 2002) and the majority of hemizygous Hccs heart specific knock out mutants as well as homozygous females die in uterus between E10.5 and E12.5 (Drenckhahn 2008). c) All Mo injection experiments generate a level of heterogeneity and therefore, the morphant pool we have analyzed necessarily includes embryos with heterogeneous levels of hccs down-regulation. Together these reasons explain our results. For all the above-mentioned reasons we cannot assess the levels of complex III activity in a homogeneous group of morphants with high levels of hccs downregulation. Nevertheless, to support our conclusions, we repeated this experiment, analyzed more embryos and obtained an highly significant (p=0.00002) decrease in complex III activity. In addition the levels of activity of additional complexes (namely complex I, II and IV) were evaluated and the data have been included in the Supporting Information Figure S4. Please, note that complex IV activity was measured using exogenous cytochrome c, and hence was unaffected in morphants.
This data have been incorporated in the text, page 9. Statistics for aberrant mitochondria was calculated and added to Supporting Information Figure S6. The figure has been modified as requested and is now the Supporting Information Figure S6.

2. The number of normal hccs-MO embryos is increased upon NAC treatment suggesting deleterious effects of an increased ROS production. Does NAC treatment suppress the activation of caspase 9 (as monitored in Fig. 6H)?

As requested we evaluated the activation of caspase-9 upon NAC treatment in morphant embryos. Our results illustrated in Figure 6 (panel E) and at page 13 indicate that NAC treatment rescues increased levels of caspase 9 activation *in vivo*.

3. Is it possible to assess cytochrome c levels in hccs-MO?

As requested, we evaluated the cytochrome c levels in total extracts and mitochondrial fractions. Our results illustrated in Figure 2G and in the Supporting Information Figure S5 have been included in the text, page 9, and indicate that hccs downregulation induces decreased levels of cytochrome c *in vivo*. This data further support the role of HCCS in MRC function.

4. The experiments in yeast (Fig. 1) appear rather disconnected from the rest of the manuscript which focusses on the medaka model. The well-performed experiments demonstrate functional conservation but otherwise are of limited novelty and therefore may be deleted from the manuscript.

As requested by the editor and reviewer #3 the figure illustrating the experiments in yeasts has been included in the Supporting information section (Supporting Information Figure S1).

Referee #2 (Other Remarks):

EMM-2012-01739 "HCCS, the Gene Responsible for MLS Syndrome, Regulates Activation of a Non-Canonical Cell Death Pathway in brain and eye explaining the disease phenotype" by Dr. A. Indrieri et al.

COMMENTS TO THE AUTHORS

In this paper, Indrieri and colleagues examine a medaka model which recapitulates the phenotype of Microphthalmia with Linear Skin lesions (MLS), an X-linked developmental disorder caused by mutations in the Holo-Cytochrome c-type synthase (HCCS) gene. The study shows that downregulation of hcc causes increased cell death via an apoptosome-independent caspase-9 activation in brain and eyes. Thereby, caspase-9 activation in the mitochondria is triggered by impairment of the mitochondrial respiratory chain and an overproduction of reactive oxygen species.

This is a useful study which indicates that HCCS is a key molecule in the development of the central nervous system that modulates a new non-canonical cell death and provides the first experimental evidence for a mechanistic link between mitochondrial dysfunction, intrinsic apoptosis and developmental disorders.

There are, however, some major and minor comments the authors may wish to consider.

Major criticisms:

1. The authors propose that increased ROS production, caused by an impairment of the mitochondrial respiratory chain triggers tissue-specific programmed cell death, leading to MLS. It would be of interest, which ROS species are increased. Please specify.

We set up the conditions to use MitoSOX that detects mitochondrial superoxide, *in vivo*. Our data demonstrated a strong increase of mitochondrial ROS in morphants. These results have been included in the text (page 13) and in Supporting Information Figure S9. To the best of our knowledge there are no other tools to evaluate which species of ROS are modified *in vivo* and our results support our hypothesis that the increased levels of ROS in our model is due to MRC impairment.

2. The yeast studies are very interesting; please confirm the results for cytochrome C expression in the medaka model (western blot analysis of cytosolic and mitochondrial fraction).

As requested, we evaluated the cytochrome c levels in total extracts and mitochondrial fractions. Our results illustrated in Figure 2G and in the Supporting Information Figure S5 have been included in the text, page 9 and indicate that *hccs* downregulation induces decreased levels of cytochrome c *in vivo*. This data further support the role of HCCS in MRC function.

Page 1, keywords: change to "... Holo-Cytochrome c-type synthase ..."
Page 3, line 9: Morleo & Franco 2009 is missing in the "References"

Page 6, line 9: change to "... MRC complexes ..."
Page 8, line 26: change to "... (10 {plus minus} 5%) ..."
Page 10, line 1: change to "... XIAP ..."
Page 10, line 4: change to "... apoptosome complex ..."
Page 10, line 19-20: change to "... caspase-8 ... caspase-9 ..."
Page 13, line 12: change to "...Holo-Cytochrome c-type synthase ..."
Page 15, line 1: change to "... complexity..."
Page 17, line 7: "...ForMedium{trade mark, serif}, UK ..." City?
Page 17, line 9: "...Carlo Erba Reagents, Italy ..." City?
Page 17, line 9: change to "... 20 g/l..."
Page 17, line 12: change to "... 33 {degree sign}C..."
Page 17, line 14: "... Cary 300 Scan spectrometer..." Company, City, Country?
Page 17, line 15: change to "... 30 {degree sign}C... 3 ml..."
Page 17, line 16: change to "... 0.1 M...10 mM..."
Page 17, last line: "... Bio-Rad Protein Assay..." Company, City, Country?
Page 18, line 4: "... Cytc, Atp4..." Company, City, Country?
Page 18, line 8: change to "... 28 {degree sign}C..."
Page 18, line 11: "... (Gene Tools, LLC)..." Company, City, Country?
Page 18, line 15: "... (Ambion)..." City, Country?
Page 19, line 5: change to "... and TUNEL assay ..."
Page 19: "... (Cell Signaling Technology)... (BD Pharmingen{trade mark, serif}) ... (Vector laboratories) ... (Roche) ... (Leica Microsystems) ..." City, Country?
Page 19, line 14, 15: change to "... 4 {degree sign}C..."
Page 19, line 16: change to "... Epon 812 ..."
Page 19, last line: change to "... Olympus Soft Imaging System GmbH, Münster, ..."
Page 20, line 1: change to "... Caspase Assays ..."
Page 20, line 5: change to "... 50 mM ... 2.5 M MgCl2"
Page 20, line 7: change to "... 100 µl ..."
Page 20, line 8: "... (Promega)..." City, Country?
Page 20, line 9: change to "... 2 h ..."
Page 20, last line: change to "... Olympus Soft Imaging System GmbH, Münster, ..."
Page 20, line 13: Ac-YVAD-CHO is a caspase-1 and caspase-4 inhibitor!
Page 21, line 1: change to "... 2 h ..."
Page 28, line 8: change to "... Western blot ..."
Figure 1B: Unit of the "Respiratory activity" is missing!
Figure 8: change "CYTOPLAMS" to "CYTOPLASM"
SUPPORTING INFORMATION Page 2, line 1: change to "... 10-400 µM ..."
SUPPORTING INFORMATION Page 2, line 3: change to "...100 ng ... 25 ng ..."
SUPPORTING INFORMATION Page 2, last line: change to "... 300 µM ..."
SUPPORTING INFORMATION Page 3, line 1: change to "... 400 µM ..." Remove the red highlights!
SUPPORTING INFORMATION Page 3, line 2: change to "... 30 µM ..."
SUPPORTING INFORMATION Page 3, line 3, 4: change to "... 100 µM ..."
SUPPORTING INFORMATION Page 3, line 10: change to "... 33 {degree sign}C ..."
SUPPORTING INFORMATION Page 3, line 20: change to "... -80 {degree sign}C ..."

SUPPORTING INFORMATION Page 3, line 21: change to "... 250 mM ... 20 mM ... 3 mM ..."
Supporting Table S1: Stated are standard deviations (SD), but in the "Materials and Methods" (Page 20) "Quantitative data are presented as the mean {plus minus} SEM" is denoted. Please explain.
Supporting Figure S2, Figure H: SEM is missing!
Supporting Figure S2, line 2: change to "... st38 ..."
Supporting Figure S2, line 8: change to "... st19 ..."

All minor criticisms indicated by the reviewer have been corrected

Referee #3 (Comments on Novelty/Model System):

This study provides convincing data about the pathological cause of the microphtalmia in MLS syndrome, an issue that has not been address before because of the lack of a good model for the disease. Although MLS syndrome is a rare disease, the study has a broader interest as it provides insights into the current knowledge of caspase-9 mediated cell death.
The study is very good and it fits to the scope of the journal. Experiments are well thought and, in general, nicely executed.
Although the study does not address the mechanistic link between increased ROS and caspase-9 activation its contribution to the understanding of the disease is significant. In my opinion a revised version of the paper addressing the comments and suggestions made should be sufficient for a cceptance of t he paper for publ ication. Suggested additional experiments to support activation of mitochondrial caspase-9 as the underlying cause of the disease are desirable but not a requisite for publication of the paper.

Referee #3 (Other Remarks):

This study focuses on MLS syndrome, an X-linked disease characterized by unilateral or bilateral microphtalmia and linear skin defects, which is caused by mutations in HCCS. This gene encodes a m itochondrial enzyme required for the maturation of Cy tc and Cytc1. To model the disease the authors used morpholinos and knocked-down the hccs gene in the medaka fish. They performed a s et of experiments and show that microphtalmia caused by dowregulation of the medaka hccs gene is the consequence of an increased developmental cell death that involves activation of caspase-9 and caspase-3/7. They also show that caspase-9-mediated cell death in the eyes of Hccs knockdown embryos does no re quire Apaf-1 and provide evidences indicating that activation of caspase-9 occurs within the mitochondria. They go on and show that production of ROS in Hccs knockdown embryos is higher than in controls. Base on these experiments, and on experiment performed in yeast strains that express human HCCS mutant alleles, they conclude that microphtalmia in MLS syndrome is due to the activation of a non-canonical mitochondrial-dependent cell death pathway.

Results present in this manuscript are novel and provide a mechanistic explanation for microphtalmia in MLS syndrome. They also provide new insights into the current knowledge of c aspase-9 mediated cell death that are of broader interest. The authors should address the following comments and questions before acceptance of the paper for publication.

Comments and questions:

1. One of the important findings of the study is the activation of caspase-9 by a mechanism that does not require the formation of the apoptosome. Based on the experiments shown in Figure 6 (D to H), the authors propose a model by which activation of caspase-9 occurs within the mitochondria. Further experimental evidences (i.e.: measurements of caspase-9 activation in mitochondrial and cytoplasmic fractions) would strengthen the proposed model.

As requested by the reviewer we measured caspase-9 activation in mitochondrial and cytoplasmic fractions from morphants and controls. The results have been included in Figure 6 (panel E) and illustrated in the results section page 13.

2. The authors cite examples of apoptosome-independent caspase-9 activation (page 11). They should also include the work by Manns and colleagues published last year in FASEB J.

Following the reviewers suggestion the work by Manns and colleagues has been cited (page 11) and added to the reference list

3. What is the contribution of mitochondrial caspase-9 activation (Apaf1-independent) in normal developmental cell death? In other words, is this non-canonical cell death pathway relevant in physiological conditions or is activated only in situations of a pathological increase of ROS? This issue should be addressed in the discussion section and the Title of the manuscript and last sentence of the Abstract modified accordingly.

Actually our data represent the first indication of a role for this non-canonical cell death pathway *in vivo*. According to our data mitochondrial caspase-9 activation indeed plays an important role in developmental cell death. There is no information on the role of this pathway in physiological conditions and this will be one of the objects of our future investigations. Recent evidences indicate that ROS should not be thought only as toxic byoproducts of aerobic metabolism as they may act as signaling molecules in the cell and thus play a central role in complex signaling network and in physiological cellular responses. In view of these consideration it is very well possible that the ROS-dependent pathway we have described may play a role in normal development and we hope to address this issue in future studies. A sentence at this regard has been added to the discussion, page 15.

4. Published data about developmental apoptosis in the Medaka nervous system (i.e. Iijima and Yokoyama; Acta Histochem. Cytochem. 40 (1): 1-7, 2007) should be cited in the manuscript and taking into account for presentation and discussion of the results.

The work by Iijima and Yokoyama has been cited in the result (page 10) and the discussion (page 14) sections.

5. Results in yeast presented in Figure 1 are not fundamental for the conclusion of the paper. This data could be moved to Supplemental information or after the results presented in Figure 5.

The figure illustrating the yeast data has been moved to the Supplemental Information section and is now the Supporting Information Figure S1.

6. Conclusions of t he results shown in Figures 1A and C a re not clear (page 7). A description of the human HCCS mutations would facilitate interpretation of the data.

A description of the human HCCS mutations has been included in the result section (page 6).

7. Developmental cell death is one of the mechanisms, but not the most common mechanism, that regulates the size of a cell population. First sentence, second paragraph, in page 4 is not correct and needs to be written again.

The sentence has been modified and now reads: "During central nervous system (CNS) development programmed cell death (PCD) represents an important mechanism to regulate the size of cell population although the regulatory mechanisms of survival/death are not fully characterized".

8. To avoid unnecessary repetitions, the paragraph describing the canonical intrinsic cell death pathway in the Introduction section (pages 4-5) should be summarized or eliminated from the Introduction section.

The paragraph describing the canonical intrinsic cell death pathway in the Introduction section has been shortened (pages 4-5)

9. Cell death in the central nervous system affects neuroepithelial progenitors as well as differentiating neurons (reviewed in Valenciano et al, 2009). First paragraph in the Discussion section needs to be written accordingly to this revision.

The first paragraph of the discussion has been revised (page 14)

10. The eye phenotype in st24 embryos injected with *hccs*-MO seems more severe than in st30 (Figures 4 C a nd D). Is the section shown in Figure 4C re presentative of t he phenotype?

Actually if each panel displaying morphants is compared to its age-matched control, the phenotype at st24 is not more severe than that observed at st30. At st30 there are still cells dying in morphants (panel D) whereas there is basically no cell death in the controls at this stage (panel B). Please note that the scale bar in panels B and D is different and thus morphants at st30 show a severe microphthalmia.

Minor issues:

- Text in histograms shown in Figures 4, 5, 6 and 7 are difficult to read.
- Figure 8 needs some modifications; schemes are not clear and letters too small.

All minor criticisms have been corrected, Figure 8 has been revised

2nd Editorial Decision 02 December 2012

Thank you for the submission of your revised manuscript to EMBO Molecular Medicine. We have now received the enclosed reports from the referees that were asked to re-assess it. As you will see the reviewers are now globally supportive and I am pleased to inform you that we will be able to accept your manuscript pending the final corrections requested by Reviewer 2. In addition please read carefully, and abide to (where you have not already done so), the important information for submitting your revised manuscript pasted below.

Please submit your revised manuscript as soon as possible and no later than two weeks from today.

I look forward to seeing a revised form of your manuscript .

Yours sincerely,

Scientific Editor
EMBO Molecular Medicine

***** Reviewer's comments *****

Referee #1:

The authors have carefully addressed my concerns on the original manuscript and I can therefore support publication of this manuscript.

Referee #2:

EMM-2012-01739-V2 "HCCS, the Gene Responsible for MLS Syndrome, Regulates Activation of a Non-Canonical Cell Death Pathway in brain and eye explaining the disease phenotype" by Dr. A. Indrieri et al.
In this now well written paper, Indrieri and colleagues examine a medaka model which recapitulates the phenotype of Microphthalmia with Linear Skin lesions (MLS), an X-linked developmental disorder caused by mutations in the Holo-Cytochrome c-type synthase (HCCS) gene. The study shows that downregulation of hcc causes increased cell death via an apoptosome-independent caspase-9 activation in brain and eyes. Thereby, caspase-9 activation in the mitochondria is triggered by impairment of the mitochondrial respiratory chain and an overproduction of reactive oxygen species.
This is a useful study which indicates that HCCS is a key molecule in the development of the central nervous system that modulates a new non-canonical cell death and provides the first experimental evidence for a mechanistic link between mitochondrial dysfunction, intrinsic apoptosis and developmental disorders.
I think the manuscript is now acceptable for publication in "EMBO Molecular Medicine" with a few minor corrections.

Minor criticisms:

Page 18, last line: change to "... Bio-Rad Protein Assay (Bio-Rad..."
Page 20, line 15: change to "... Bio-Rad Protein Assay (Bio-Rad, "
Page 24, line 3: change to "...and B.F. ..." "...P.B. and B.F. designed ..."
Page 24, line 4: change to "...and G.C. ..." "...P.G., I.F., J.Q. designed ..."
Page 24, line 6: change to "...and B.F. ..."
Page 28, line 28-29: Please cite "Morleo and Franco 2009" correctly.
Page 33, line 22: change to "... significantly..."
Page 34, line 12: change to "... Apaf1-independent..."

Referee #3:

The authors adequately addressed all my concerns including in the new version of the manuscript additional experiments and revisions to the text and figures.

2nd Revision - authors' response 03 December 2012

On behalf of my co-authors please find enclosed our revised manuscript titled: "HCCS, the Gene Responsible for MLS Syndrome, Regulates Activation of a Non-Canonical Cell Death Pathway in brain and eye explaining the disease phenotype", by Indrieri et al.

We modified the text according to the suggestions requested by reviewer 2 and we modified the text and the figures taking into account the EMBO instructions.

We hope you find the manuscript suitable for publication in EMBO Molecular Medicine and look forward to hearing from you at your earliest convenience.
