# Supplementary material for: The impairment of HCCS leads to MLS syndrome by activating a non-canonical cell death pathway in the brain and eyes
Source: EMBO Mol Med. 2013 Jan 22;5(2):280–93. doi: 10.1002/emmm.201201739 (PMC3569643; doi:10.1002/emmm.201201739)
Supplement: Supplementary file 2 [file emmm0005-0280-SD2.pdf]

SUPPORTING INFORMATION

The impairment of HCCS leads to MLS syndrome by activating a non-canonical cell death pathway in the brain and eyes

Alessia Indrieri, Ivan Conte, Giancarlo Chesi, Alessia Romano, Jade Quartararo, Tatè Rosarita, Daniele Ghezzi, Massimo Zeviani, Paola Goffrini, Ileana Ferrero, Paola Bovolenta, Brunella Franco

TABLE OF CONTENTS

Supporting material and methods

Supporting references

Supporting Information Figures (S1-S9) and Supporting Information Table S1

SUPPORTING MATERIAL AND METHODS

Identification of the hccs gene

We identified the medaka orthologous gene by screening the sequences available through the medaka genome project (available at http://www.ensembl.org/Multi/blastview) with the human HCCS and mouse Hccs protein sequences (NP_005324 and NP_032248 respectively). The entire *hccs* coding sequence including part of the 5'UTR region was isolated by RT-PCR amplification from a cDNA derived from a pool of medaka embryos at different stages using specific primers listed below.

MO and mRNA Injections

hccs-MO and hccs-MO2 were injected at 10-400 μM, inducing a dose-dependent phenotype. Sequences and selected working concentrations for all MOs are listed below. A total of 100 ng of *hccs* and *Bcl-xL* mRNAs and of 25 ng of GFP and RFP mRNAs were used for each injection.

To ensure the specificity of the hccs-MO, we performed appropriate control experiments (Eisen & Smith, 2008). The control-MO was a MO with the substitutions of five bases (indicated in red below) with respect to hccs-MO. The phenotype was rescued by co-injecting the MO and the *hccs* mRNA, demonstrating that all the defects observed in the injected embryos were due to specific downregulation of *hccs* (Supporting Figure S3C). Activation of p53 can represent a non-specific off-target effect of some MOs (Robu et al, 2007). In this case the phenotype can be rescued by co-injection of a MO against p53 (Eisen & Smith, 2008). We thus co-injected hccs-MO with *p53*-MO (Conte et al, 2010) and no amelioration of the phenotype was observed (Supporting Figure S3D) indicating

that in this case the activation of p53 is not involved in the observed phenotype. Efficiency for *hccs*-MO was measured as the ability of interfering with GFP expression using a reporter construct, as previously reported (Esteve et al, 2004). The pCS2/5'UTRhccs::GFP reporter plasmid was constructed by cloning the complementary region of the MO in frame with the GFP coding sequence (Supporting Figure S3E). Its inhibitory efficiency was measured by quantification of GFP intensity using the ImageJ analysis software (Supporting Figure S3F-H).

Sequences of MOs and oligonucleotides used to generate constructs and RNA ISH templates

hccs-MO (300 µM) 5'-AGGTGTAGACGCAGAAGCGCCCATC-3'

control-MO (400 µM) 5'-ATGTGTAAACGCATAAGCTCCCATT-3'

hccs-MO2 (30 µM) 5'-TGAAGTCAGGAACGTACCATGTTAG-3'

Apaf1-MO (100 µM) 5'-CTTCAGGCAAGTCACCTCCGACCAT-3'

p53-MO (100 µM) 5'-CGGGAATCGCACCGACAACAATACG-3'

hccs-Fw 5'-TGCCGGTCGGTGGGTCCTTTG-3'

hccs-Rv 5'-CCAGTCTGCTGAAGCGCTGCAC-3'

hccs-degenerate-Fw 5'-TATGGGAGCGAGTGCTTCCACACCTGC-3'

Chronological life span and survival to H_2O_2 treatment

For chronological life span yeasts strains were grown until stationary phase and incubated at 33 °C in flasks with a volume/medium ratio of 5:1, shaking at 200 rpm. Viability was measured over time by plating serial dilutions of the cultures onto YPD plates (0,5% yeast extract, 1% peptone, 2% glucose, 2% agar) and counting colony-forming units after

three days of growth. The yeast sensitivity to H_2O_2 was assayed by disc diffusion. 20 ml of YNB agar medium were added to the plates; once this first layer solidified was added a second layer (10 ml YNB) containing a total amount of 10^6 cells from an exponential phase culture. Discs (Whatman™) containing H_2O_2 (1% to 10%) were used for disc diffusion assay. Plates were incubated for 3-4 days and inhibition halos were measured.

Biochemical analysis of MRC complexes activity

Embryos harvested at st19-24 (80 per sample) were dechorionated and quick frozen in liquid nitrogen then stored at -80 °C until ready for use. The embryos were resuspended in 200 ul buffer (320 mM sucrose, 1 mM EDTA, 10 mM TrisHCl pH 7.4) and snap-frozen and thawed three times before enzyme measurements. Biochemical analyses were performed on the 800 x g supernatants; MRC complexes I, II, III and IV activities were measured spectrophotometrically as described (Ghezzi et al, 2011) and normalized for citrate synthase activity.

SUPPORTING REFERENCES

Conte I, Carrella S, Avellino R, Karali M, Marco-Ferreres R, Bovolenta P, Banfi S (2010) miR-204 is required for lens and retinal development via Meis2 targeting. *Proc Natl Acad Sci U S A* **107:** 15491-15496

Eisen JS, Smith JC (2008) Controlling morpholino experiments: don't stop making antisense. *Development* **135:** 1735-1743

Esteve P, Lopez-Rios J, Bovolenta P (2004) SFRP1 is required for the proper establishment of the eye field in the medaka fish. *Mech Dev* **121:** 687-701

Ghezzi D, Arzuffi P, Zordan M, Da Re C, Lamperti C, Benna C, D'Adamo P, Diodato D, Costa R, Mariotti C, Uziel G, Smiderle C, Zeviani M (2011) Mutations in TTC19 cause mitochondrial complex III deficiency and neurological impairment in humans and flies. *Nat Genet* **43:** 259-263

Robu ME, Larson JD, Nasevicius A, Beiraghi S, Brenner C, Farber SA, Ekker SC (2007) p53 activation by knockdown technologies. *PLoS Genet* **3:** e78

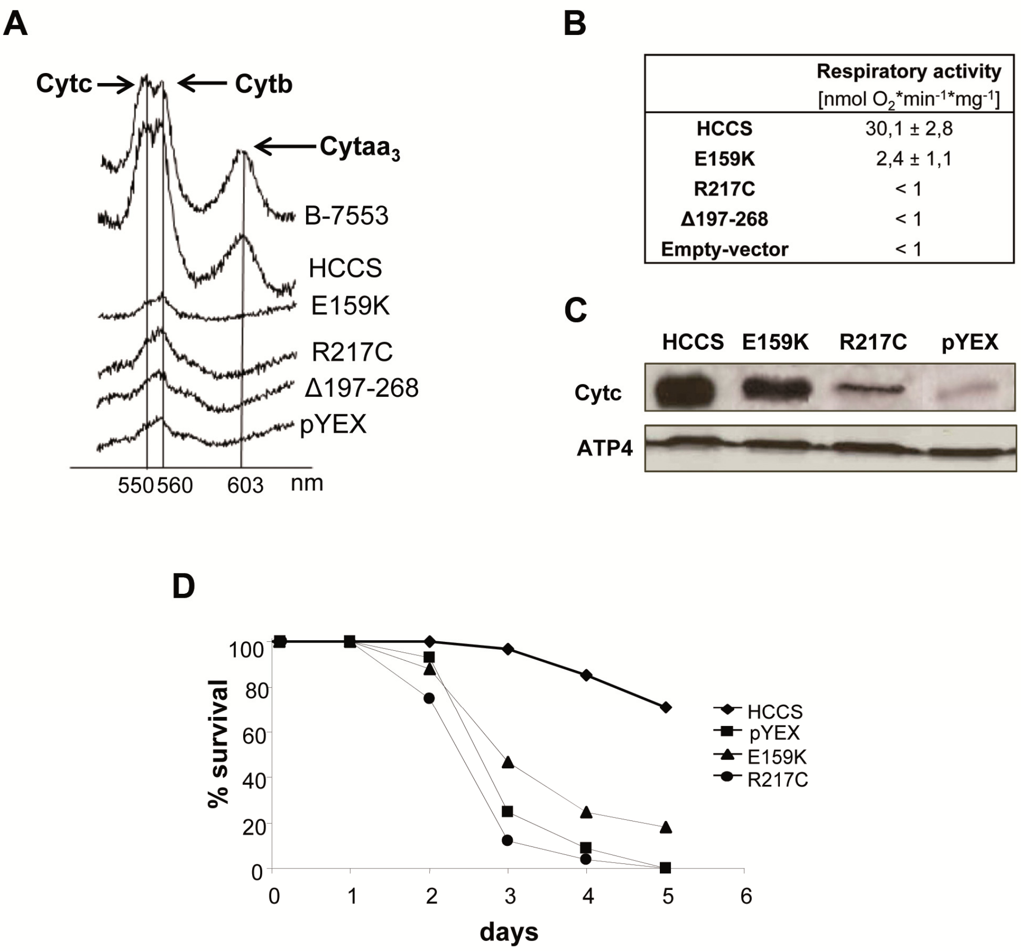

Supporting Information Figure S1. Effects of HCCS deficiency on mitochondrial respiratory function in yeast. (A) Cytochromes absorption spectra of the B-8025-Δ*cyc3* yeast strain transformed with the pYEX empty vector (pYEX), the human wt *HCCS* (HCCS) and the *HCCS* mutated alleles (E159K, R217C, Δ197-268). The B-8025-Δ*cyc3* strain transformed with *HCCS* mutant alleles and pYEX empty vector shows a pronounced reduction of the absorption peak corresponding to Cytc and Cytaa$_3$. (B) Respiratory activity of transformed yeast strains expressed as oxygen consumption [nmol O_2*min^{-1}*(mg dry weight)$^{-1}$]. Each value is the mean of three independent experiments ± the standard deviation. (C) Western blot analysis of Cytc in transformed yeast strains mitochondrial fractions. (D) Chronological life span (CLS) of the B-8025-Δ*cyc3* yeast strain transformed with wt *HCCS* (HCCS) and pathological alleles (E159K and R217C) and with the pYEX empty vector (pYEX).

```
                          10        20        30        40        50        60
                  ....|....|....|....|....|....|....|....|....|....|....|....|
HCCS              MGLSPSAPAVAVQASN------------------ASASPPSGCPMHEGKMKGCPVNTEPS
hccs              MGASASTPAAVTVQAEAVAAAPQGRSVHQEVQPVQKASPQPECPMHQTPP----VKASP-
Clustal Consensus ** * *:**...  ::                   .***   ****:       *::.*

                          70        80        90       100       110       120
                  ....|....|....|....|....|....|....|....|....|....|....|....|
HCCS              GPTC-EKKTYSVPAHQERAYEYVECPIRGTAAENKENLDPSNLMPPPNQTPAPDQPFALS
hccs              PPECPMHKAEPGPAHQNQAYQFVECPMRAAAG-VRSDIDPANMMPPPNQTPAPDQPFPLS
Clustal Consensus  * *  :*:   ****::**::****:*.:*.  :.::**:*:************** **

                         130       140       150       160       170       180
                  ....|....|....|....|....|....|....|....|....|....|....|....|
HCCS              TVREESSIPRADSEKKWVYPSEQMFWNAMLKKGWKWKDEDISQKDMYNIIRIHNQNNEQA
hccs              VVREESTIPRHGTEKNWVYPSEQMFWNAMLRKGWRWREDDLAAPDMTNIIQIHNKNNEQA
Clustal Consensus .*****:***  :**:**************:***:*:::*::  ** ***:***:*****

                         190       200       210       220       230       240
                  ....|....|....|....|....|....|....|....|....|....|....|....|
HCCS              WKEILKWEALHAAECPCGPSLIRFGGKAKEYSPRARIRSWMGYELPFDRHDWIINRCGTE
hccs              WQEILKWEALHAGECPCGPTLKRFGGKAKEFSPRARLRHWMGYELPFDRHDWIIDRCGKE
Clustal Consensus *:**********.******:* ********:*****:* ***************:***.*

                         250       260       270       280
                  ....|....|....|....|....|....|....|....|....|..
HCCS              VRYVIDYYDGGEVNKDYQFTILDVRPALDSLSAVWDRMKVAWWRWTS
hccs              VRYVIDYYDGEINKDTYHFSILDVRPAFDSLGAVWDRMKVAWWRWTS
Clustal Consensus **********   :. *:*:*******:***.***************
```

Supporting Information Figure S2. ClustalW multiple alignments of the human (HCCS) and the medaka (hccs) amino acid sequences. The medaka *hccs* transcript, [Accession Number: HE646677] codifies for a protein of 281 amino acids and shows 64% identity with the human HCCS protein.

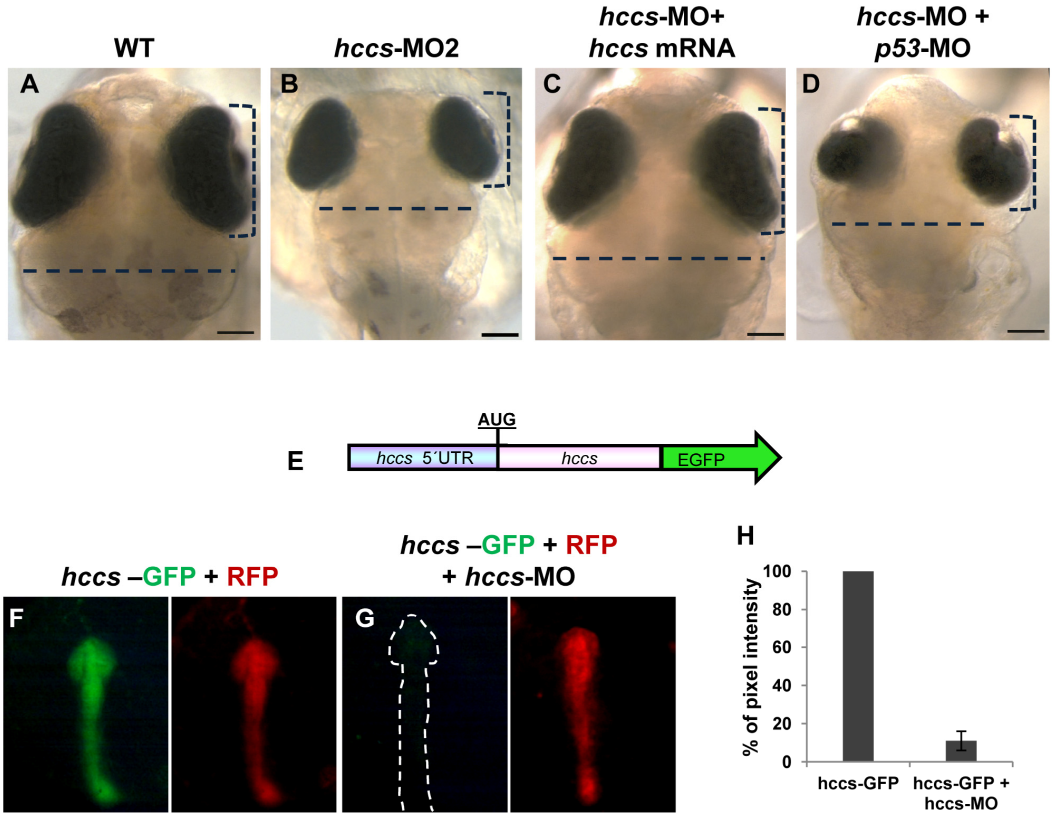

Supporting Information Figure S3. Controls of *hccs*-MO injections. Bright-field dorsal views of wt (A), *hccs*-MO2 (B), *hccs*-MO + *hccs* mRNA (C), *hccs*-MO + p53-MO (D) injected embryos at st38. The injection of the *hccs*-MO2 induces in the fish the same phenotype observed after injection of *hccs*-MO (B). The over-expression of *hccs* mRNA fully rescued the phenotype induced by *hccs*-MO (C). No amelioration of the phenotype was observed in embryos co-injected with *hccs*-MO and *p53*-MO indicating that the activation of p53 is not involved in the phenotype observed in *hccs*-morphants. Scale bars: 100 µm. (E) Schematic representation of the *hccs*-GFP construct used to test the efficiency of the MO: the construct contained the 5′ portion of *hccs* fused in frame with the GFP coding region. (F–G) Dorsal views of st19 representative embryos injected with RFP and *hccs*-GFP mRNA alone (F) or in association with *hccs*-MO (G). Note how *hccs*-MO significantly inhibits GFP expression. (H) Percentage of MO inhibition quantified by GFP/RFP intensity using the ImageJ analysis software (Error bars are SEM).

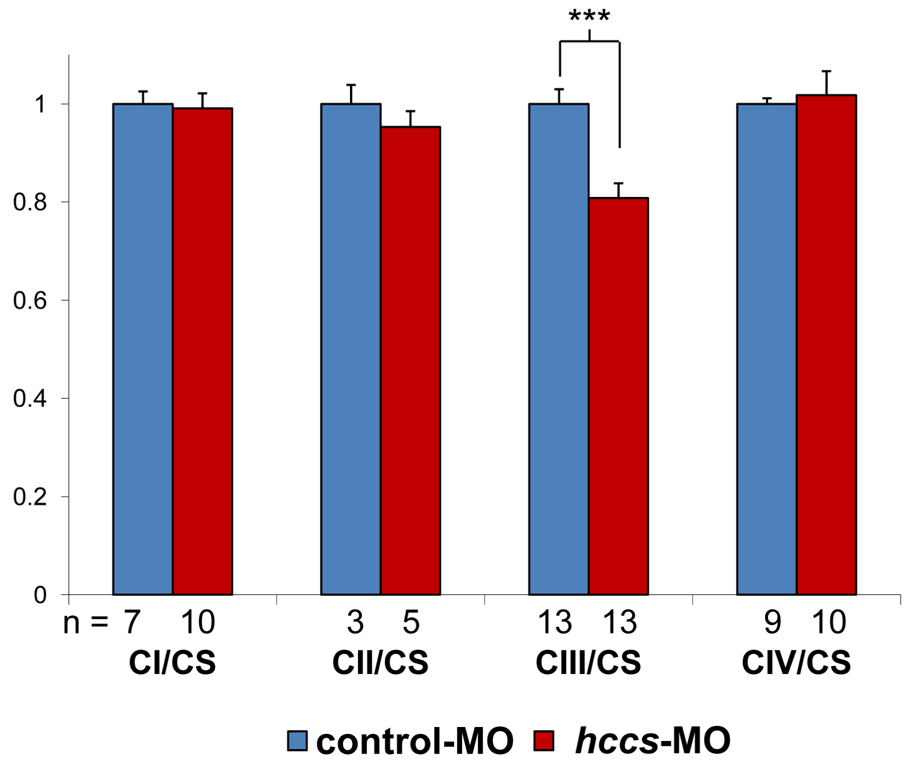

Supporting Information Figure S4. Activity of MRC complexes in *hccs*-morphant embryos. Specific activities of MRC complexes normalized to that of citrate synthase (CS). Values represent means of n samples. Each sample represents a group of 80 embryos. Error bars are SEM; *** p=0.00002 unpaired, two-tailed Student's t-test.

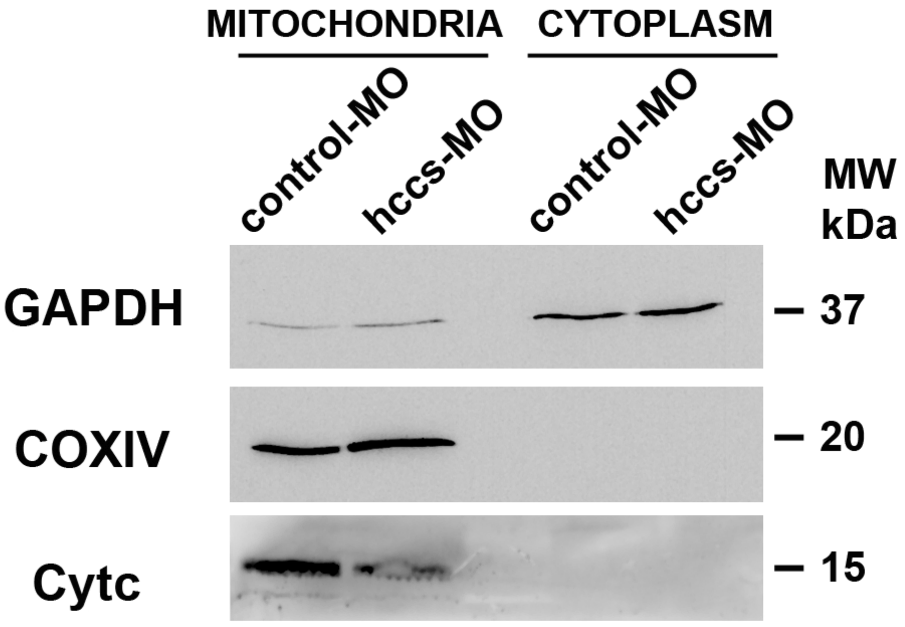

Supporting Information Figure S5. Analysis of Cytc levels in *hccs*-morphants. Western Blot analysis of mitochondrial and cytoplasmic fractions of control-MO and *hccs*-MO injected embryos at st24. GAPDH was used to detect cytoplasmic contamination and COXIV as loading control of the mitochondrial fraction. Note how morphant embryos show a decreased level of Cytc in the mitochondria.

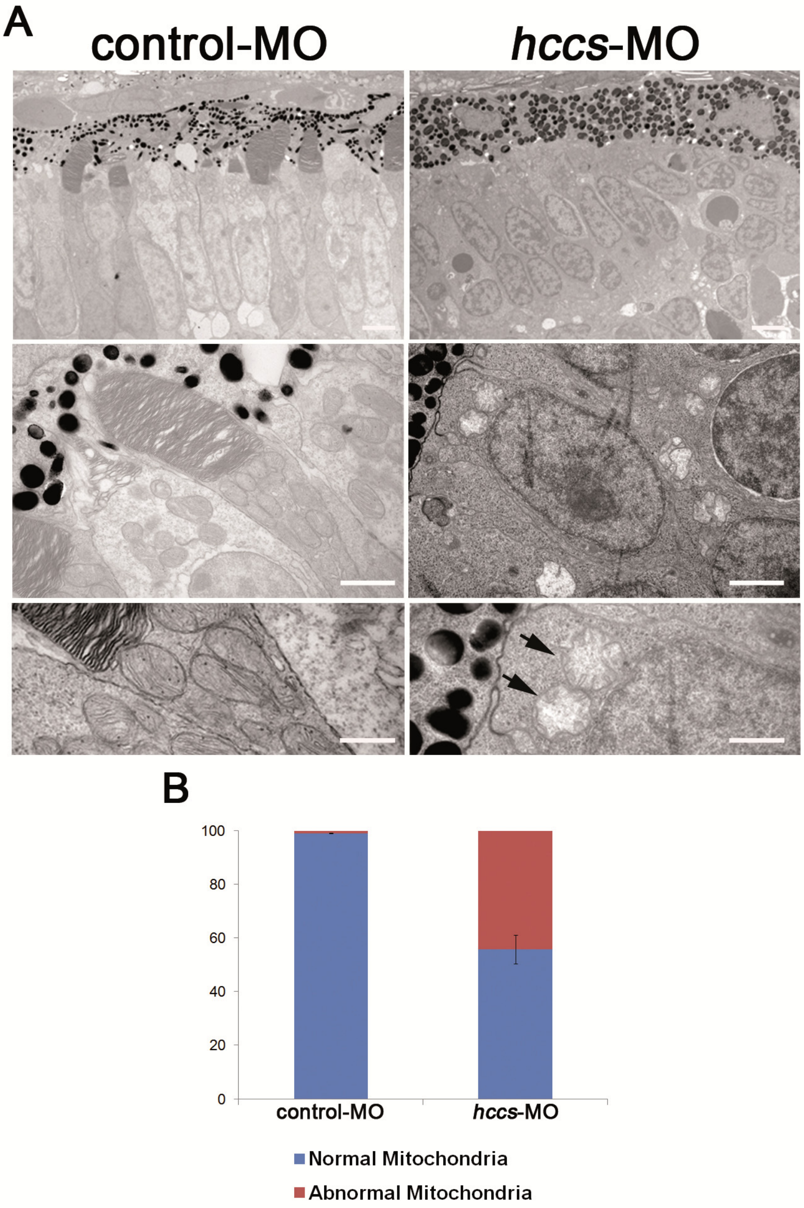

Supporting Information Figure S6. TEM analysis of mitochondrial morphology in hccs-deficient embryos. (A) Upper panels: retinal sections of control-MO and *hccs*-MO injected embryos at st38. Scale bars 5000 nm. Middle panels: mitochondria in photoreceptor cells. Scale bars 1000 nm. Bottom panels: note mitochondria, with abnormal morphology, showing internal disorganization of the cristae (black arrows) in morphants compared to controls. Scale bars 500 nm. (B) Quantification of abnormal mitochondria. Error bars are SEM; n=3 embryos, p= 0,009, unpaired, two-tailed Student's t-test.

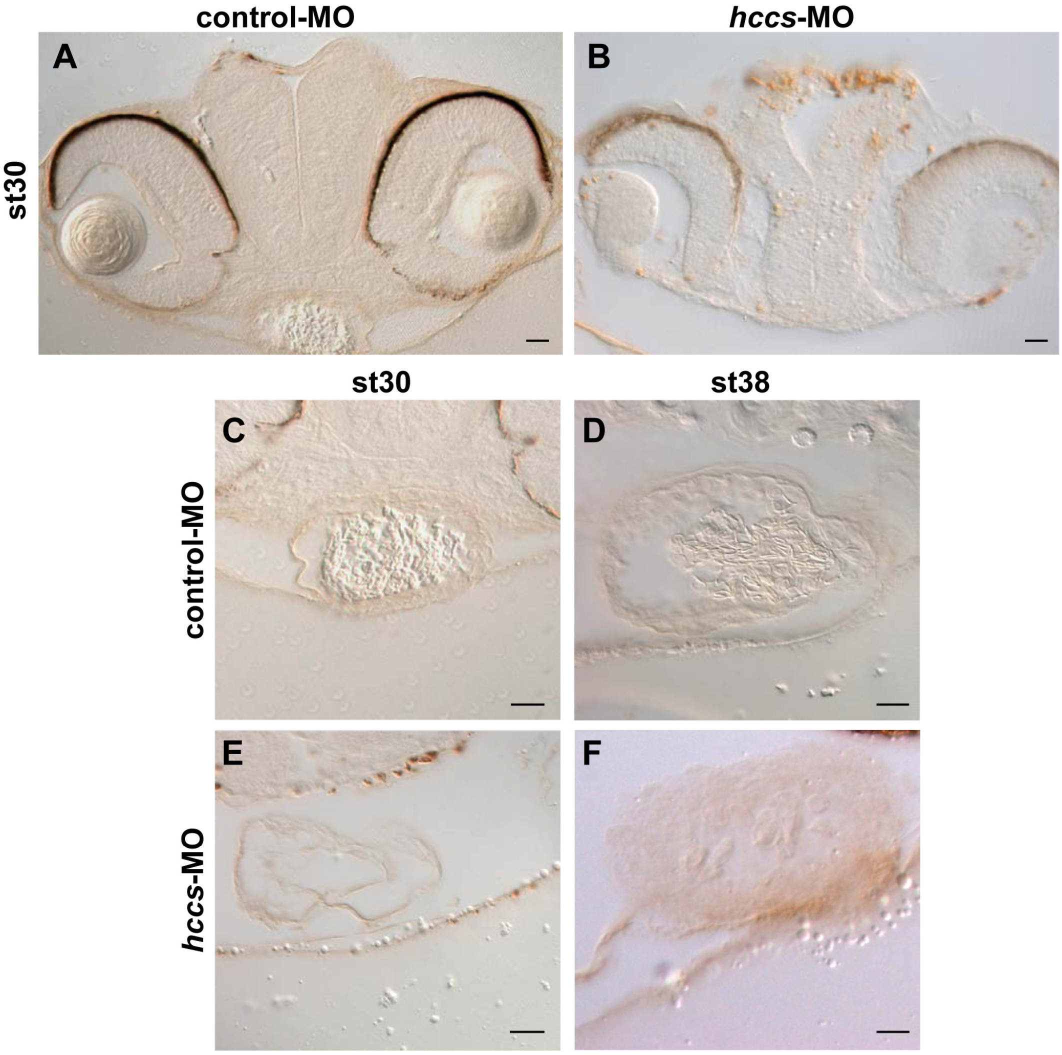

Supporting Information Figure S7. Cell death analysis in the CNS and heart of hccs-deficient embryos. (A-B) TUNEL assays on frontal sections of embryos injected with control-MO (A) and *hccs*-MO (B) at st30 showing an increased cell death in the CNS of morphants. (C-F) TUNEL assay on heart: frontal section of control-MO (C-D) and *hccs*-MO injected embryos (E-F) at st30 and st38. No alteration in programmed cell death was detected in the heart of morphant embryos. Scale bars: 20 μm.

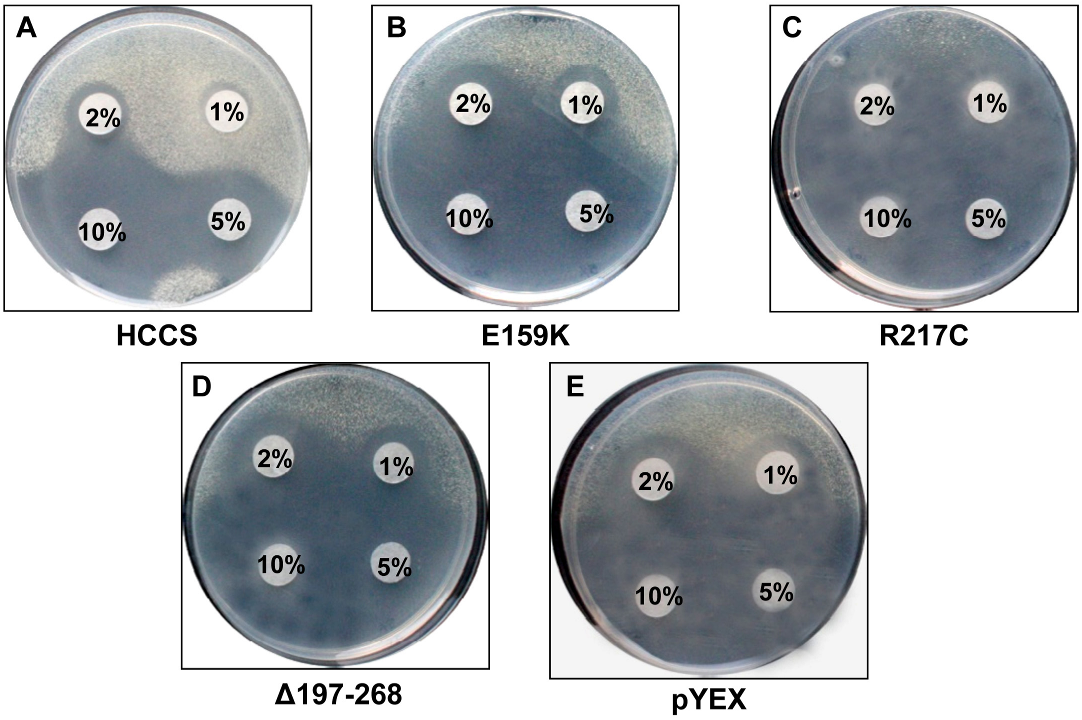

Supporting Information Figure S8. **Sensitivity to ROS of the cyc3-deficient yeast strain.** The sensitivity to H_2O_2 of the B-8025-Δ*cyc3* yeast strain transformed with wt *HCCS* (A) *HCCS* mutated alleles (B-D) and with the pYEX empty vector (E) was measured as halos of growth inhibition around the paper disk containing the substance at different concentration.

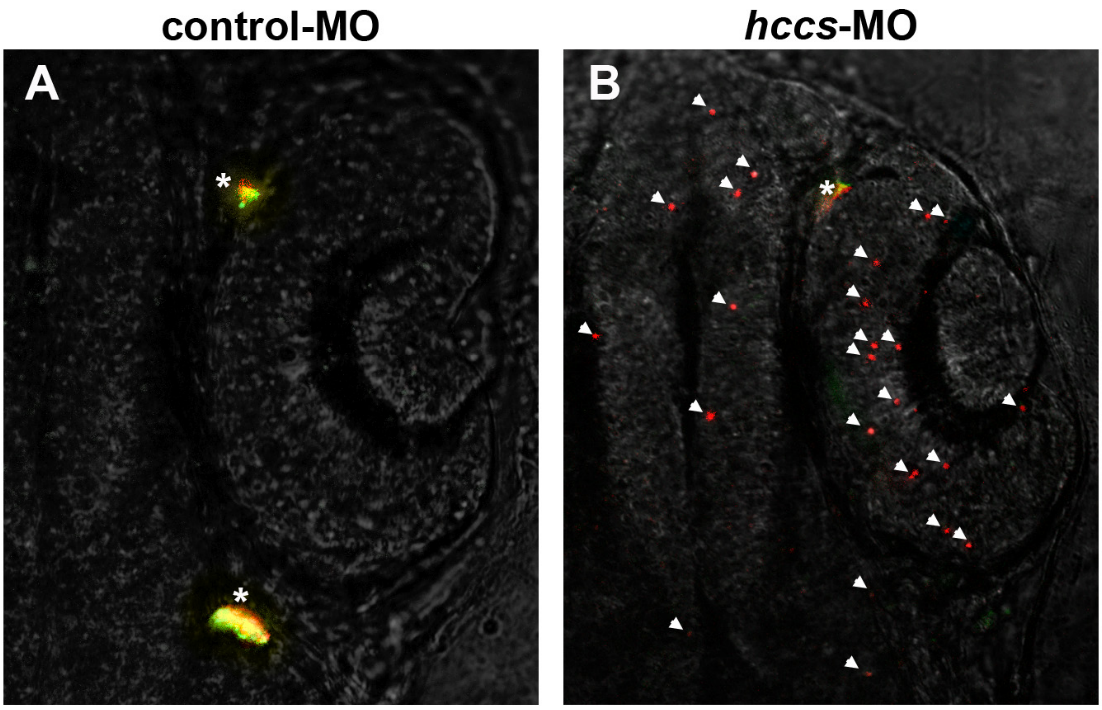

Supporting Information Figure S9. MitoSOX labeling of oxidative stress. Mitochondrial superoxide increase was detected using MitoSOX (red spots indicated by arrowheads) in living embryos at st24. Asterisks indicate auto-fluorescence of melanophores. (A) Representative image of control-MO injected embryos and of *hccs*-MO injected embryos (B). Note how labeling reveals higher level of mitochondrial superoxide in morphants compared to controls.

		Frequency of morphological phenotype (% ± SD) of n					
**Name (n)**	**Concentration**	**Microphthalmia**	**Coloboma**	**Microcephalia**	**Heart defects**	**Anophthalmia with trunk defects**	**Lethality**
hccs -MO (280)	30µM	25±5	5±2	20±5	15±5	0±0	0±0
hccs -MO (300)	100µM	55±7	18±2	45±5	40±5	8±3	5±3
hccs -MO (3000)	300µM	70±5	35±5	65±6	68±5	20±5	10±5
hccs -MO (380)	400µM	60±7	35±5	65±6	65±5	20±5	20±5
hccs -MO2 (350)	10µM	35±3	3±1	20±8	25±5	15±5	10±5
hccs -MO2 (400)	30µM	60±9	35±5	35±5	40±5	20±5	20±5
hccs -MO2 (360)	100µM	50±10	25±5	20±5	45±5	30±5	20±5
control-MO (580)	30µM-400µM	0±0	0±0	0±0	0±0	0±0	0±0
hccs -MO/ *hccs* mRNA (480)	300µM/100ng	7±2	0±0	5±2	3±1	0±0	0±0
hccs -MO/ *p53* -MO (580)	300µM/100µM	70±5	35±5	65±6	68±5	20±5	10±5
hccs -MO1 *Apaf1* -MO (760)	300µM/100µM	60±5	35±5	65±6	68±5	20±5	20±5
hccs -MO/ *Bcl-xL* mRNA (780)	300µM/100ng	20±5	10±0	18±3	20±1	0±0	5±0

n, number of injected embryos; SD, standard deviation

Supporting Table S1. Frequencies of morphological phenotypes observed after single or combined injections of MOs and mRNAs
